# A Pilot Study on Video Game Training Effects on Visual Working Memory: Behavioral and Neural Insights

**DOI:** 10.3390/brainsci15020153

**Published:** 2025-02-04

**Authors:** Héctor Hugo Alfaro-Cortés, Sulema Torres-Ramos, Israel Román-Godínez, Vanessa Doreen Ruiz-Stovel, Ricardo Antonio Salido-Ruiz

**Affiliations:** 1 División de Tecnologías para la Integración Ciber-Humana, Centro Universitario de Ciencias Exactas e Ingenierías, Universidad de Guadalajara, Guadalajara 44100, Mexico; hugo.alfaro@alumnos.udg.mx (H.H.A.-C.); sulema.torres@academicos.udg.mx (S.T.-R.); israel.roman@academicos.udg.mx (I.R.-G.); 2 Instituto de Neurociencias, Centro Universitario de Ciencias Biológicas y Agropecuarias, Universidad de Guadalajara, Guadalajara 44100, Mexico; vanessa.ruizstovel@academicos.udg.mx

**Keywords:** cognitive video game training, behavioral correlates, neural correlates, visual working memory, Negative Slow Wave, decision trees

## Abstract

**Background/Objectives:** Recent research suggests that video games may serve as cognitive training tools to enhance visual working memory (VWM) capacity. However, the effectiveness of game-based cognitive training remains debated, and the underlying neural mechanisms, as well as the relationship between training efficacy and game design factors, are unclear. This study aimed to evaluate the impact of video game training on VWM capacity and explore its neural correlates. **Methods:** Two groups underwent 56 daily 20 min training sessions with two distinct video games targeting different cognitive skills: a reaction-time training game and a VWM-specific training game. Behavioral assessments included accuracy, hit response times, correct rejection response times, and Cowan’s K values. Neural correlates were measured through Negative Slow Wave (NSW) activity using EEG. Decision tree classification analyses were applied to NSW data across sessions and set sizes to identify patterns linked to VWM capacity. **Results:** Preliminary results are that both groups showed improvements in behavioral measures (accuracy, response times, and Cowan’s K values). NSW analyses revealed a main effect of set size in both groups, and classification results indicated that NSW patterns differed between groups, across sessions, and set sizes, supporting the relationship between NSW and VWM capacity. **Conclusions:** These findings contribute to understanding NSW as a neurophysiological correlate of VWM capacity, demonstrating its plasticity through video game training. Simple video games could effectively enhance behavioral and neural aspects of VWM, encouraging their potential as accessible cognitive training tools.

## 1. Introduction

Over the past two decades, video games have garnered significant attention for their potential cognitive benefits, particularly in mitigating the natural decline in cognitive performance associated with aging. Research has explored how video game training might slow or reverse this decline, yielding mixed results [1,2]. While some studies highlight the positive impact of video games on cognitive skills, citing their engaging and immersive nature [3,4,5], others question the reliability and replicability of these findings, especially in domains like visual working memory (VWM) [6,7].

VWM, a critical cognitive function, facilitates higher-order processes like attentional control and decision-making [8,9]. Its capacity, defined by the number of objects temporarily held in memory, is often measured using the change detection paradigm [10]. This method estimates VWM capacity and behavioral metrics, such as accuracy and response time, which correlate with memory efficiency [11].

Electrophysiological measures, including Event-Related Potentials (ERPs), provide valuable insights into the neural mechanisms underlying VWM. Notable ERP components associated with VWM are the Contralateral Delayed Activity (CDA), which reflects sustained memory retention, and the Negative Slow Wave (NSW), linked to the encoding of visual stimuli [12,13]. Studies have shown that video game training enhances VWM capacity and modifies these ERP components, suggesting neural plasticity and improved attentional control [5,14].

Given the growing interest in combining behavioral and neural data, machine learning (ML) has emerged as a promising tool for analyzing EEG patterns associated with cognitive training [15,16]. This study hypothesizes that video game-based training will enhance VWM capacity, accuracy, and response time in non-gamers, with corresponding changes in ERP components, particularly the NSW. We aim to integrate ERP measures with ML models to predict post-training performance, offering a comprehensive understanding of the neural correlates of cognitive improvement.

## 2. Materials and Methods

### 2.1. Participants

Participant recruitment began through an online survey among the undergraduate engineering Mexican students (from computer science and biomedical engineering programs) at the University of Guadalajara. A general questionnaire was used to ensure that interested volunteers met the inclusion criteria: normal color vision, no neurological or motor illnesses, not being average video game players (defined as playing less than 15 h per week [17]), and having a mobile device compatible with the simple video games used for training. The study sample consisted of 24 right-handed participants (16 males and 8 females) aged between 19 and 29 years (M = 22.83, SD = 2.33). Participants were randomly assigned to the visual working memory video game training group (VWM group) (mean age of 22.58 years old, SD = 1.93) or to the reaction-time video game training group (RT group) (mean age of 23.08 years old, SD = 2.82). Each group comprised 12 participants. Although the sample size is relatively small, which may limit the generalizability of the results, it is sufficient for a pilot or exploratory study. The random assignment of participants to the training groups helps control for extraneous variables, ensuring that observed differences can be attributed to the type of training received. Additionally, the sample composition, including a balanced gender distribution and an appropriate age range, is representative for the specific purposes of this study. The experimental protocol was reviewed and approved (CI-02221) by the Research Ethics Committee of the Centro Universitario de Ciencias de la Salud (CEI-CUCS), ensuring the experiment was conducted in accordance with the Declaration of Helsinki. All participants provided written informed consent before enrolling in the study. None of the recruited participants dropped out before completing the study. Although data on socioeconomic status were not collected, we acknowledge that these factors could influence the generalizability of our results. Future studies could benefit from including such information to provide a more comprehensive characterization of the participant sample.

### 2.2. Experimental Design

We studied behavioral and electrical brain activity changes related to a simple video game training in 24 non-average video game players using a change detection paradigm. Due to our longitudinal design, the same participants performed a change detection task in three sessions (pre-, mid-, and post-training): one before the training began, which served as a behavioral and an electrical brain activity baseline, followed by the second one, performed after twenty-eight days of training—this one allowed us to observe the changes related to the first levels of difficulty of the video games—and finally, the last one, occurring after fifty-six days of consecutive training.

#### 2.2.1. Video Game Training

In order for the participants to train, a mobile application was designed with the video games, which was installed on each participant’s mobile phone. The VWM group played a video game expressly designed and implemented for VWM capacity enhancement [18]. This video game is based on the CDTs, which have been used previously to train VWM capacity and precision [19,20,21]. Subjects were required to actively maintain visual information during certain time intervals in order to accomplish the objective of the ongoing task [22]. Additionally, the video game contained attractive visual elements, background music, an avatar controlled by the participant, score tables, and progressive difficulty changes.

**Figure 1:**
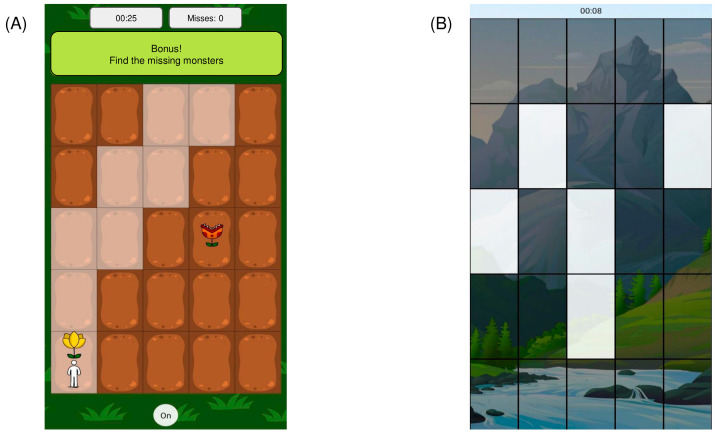
Designed video games. (**A**) Video game designed to train VWM capacity. (**B**) Reaction time video game.

The objective of the game was to guide an avatar from an initial position to a target position while avoiding all obstacles along the way. At the start of the game, a garden that represents the map through which the participant must lead the avatar is presented. Subsequently, obstacles to be avoided appear, represented by a cartoon carnivorous plant (each obstacle remains on screen for 160 ms). The participant must memorize the positions of each of the plants to ensure that he will plan a safe route for the avatar to reach its destination. After all obstacles disappear from the screen, the target position (represented by a cartoon flower) is revealed, and the participant must then press the screen of their smartphone to guide the avatar to the flower. The avatar and the flower could appear in any available space as long as they were separated by two spaces in any direction. At the end of the game, the participant had the opportunity to earn extra points by remembering and indicating on the map where the carnivorous plants they had managed to avoid were located (Figure 1A).

The difficulty of the game changed progressively but not adaptively; this means that the difficulty changed in a non-individualized way, but rather as a function of time. Each training week, the difficulty was modified by increasing the number of possible spaces available for the plants to appear and the number of plants to memorize (Week 1: 16 available spaces and 2 plants, Week 8: 25 spaces and 8 plants). There was no time limit for the trials of this game.

Furthermore, the RT group played a video game that was designed based on a simple reaction cognitive task [23] that did not involve VWM processes. It did, however, share game design elements such as background music, score tables, and progressive difficulty increments. The objective of the response game was to tap on the blank spaces that appeared on the screen as quickly and accurately as possible (Figure 1B). The difficulty of this game changed in the number of blank spaces that appeared on the screen every second, the number of possible spaces for the appearance of blank spaces, and the time they remained on the screen before disappearing if they were not tapped (Week 1: 1 to 3 blank spaces per second, 16 possible spaces positions, keys remain on screen for 2 s; Week 8: 4 to 5 blank spaces per second, 25 possible spaces, blank spaces remain on screen for 1 s). Trials for this video game lasted 1:35 min. This sort of video gaming activity performed by the RT group allows us to attribute the VWM group changes to training with the memory video game and to rule out possible non-specific generalized video game training effects, such as an improvement in visuomotor speed and sustained attention.

Participants were instructed to play their assigned video game for 20 min daily for fifty-six consecutive days (a total of 18.6 game hours). Participants were randomly assigned to each group (VWM group and RT group). Also, they were informed of the group to which they belonged and of the different experimental and control conditions from the beginning of the study. Likewise, all participants were informed of the objectives of the study before agreeing to participate.

The duration of the training was established based on similar previous studies that have observed changes in VWM when training with video games [5,24,25]. The participants played their respective games on their mobile phones using our app which automatically tracked their training time. Using the percentage of the total training time completed as a criterion, we excluded from the analyses 6 participants (3 from the VWM group and 3 from the RT group) that did not complete at least 85% of the training (15.87 h). The remaining 9 participants in the VWM group trained for an average of 17.72 h (SD = 1.16), while the RT group participants trained for 18.02 h on average (SD = 0.92). There were no significant group differences in terms of total training time.

Both video games showed a summary of the participants’ daily scores, including the highest one. Also, as a motivational incentive, the mobile app sent daily notifications to report the highest scores of the day. The participants were informed that, based on the scores, those who achieved the highest one at the end of the first and second months of training would be rewarded.

#### 2.2.2. Change Detection Task

**Figure 2:**
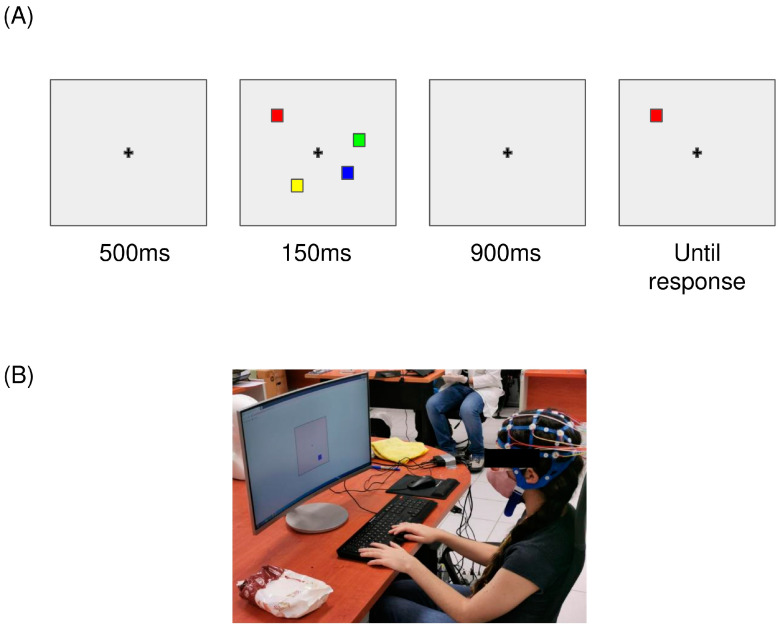
Designed change detection task and experimental setup. (**A**) Change detection task. It consist on a fixation cross to guide the eyes of the participant into the center of the screen and a set of squares for which their color and position should be memorized. (**B**) Experimental setup.

The change detection task was designed based on tasks used in previous studies that estimated VWM capacity [5,26]. To perform the participants’ evaluations, we designed, implemented, and launched a stimulus presentation software and an ad hoc Web System. The stimuli in the change detection task consisted of a set of colored squares (1.46 cm × 1.46 cm) that appeared on a gray background (13.22 cm × 13.22 cm), along with a cross-shaped fixation point at the center of the screen. The color of each square was randomly assigned, without repetition, from eight possible colors: green, red, blue, yellow, black, white, purple, and orange. The positions of the squares were also randomized on each trail. Each trail began with the presentation of the fixation cross during 500 ms (a preparation interval), and then an array of two, four, six, or eight squares was presented for 150 ms (memory set), followed by a 900 ms visual information retention interval, during which the colored squares were no longer visible. Finally, a second array (test set) was presented and remained on the screen until the participant entered their response (Figure 2A). This set was composed of a single square, randomly selected from the memory set, whose color could vary but whose position could not. The participant had to evaluate whether the color displayed matched the color seen in the memory set at that particular position.

Participants completed a total of 200 trials, 50 for each set size; the array size progressively increased, starting with two squares. Before starting the task and EEG recording, the participants performed 10 two-square training trials in order for the participant to become familiar with the task instructions. In half of the trials, the color of the square in the memory set and the test set was exactly the same. For the other half, the color of the square presented in the test set differed from the one presented in the memory set. Participants were instructed to indicate whether both arrays presented were the same or not by pressing a specific key for each answer. On every trial, participants received immediate visual feedback for both correct and incorrect responses. The cross-shaped fixation point blinked in a green color (for correct responses) or a red color (for incorrect responses).

Four dependent measures were obtained from the CDT: hits (responses when the participant correctly identifies a change) and correct rejections (participant correctly identifies that there has been no change), and their counterparts, misses (responses when the participant misidentifies a change) and false alarms (participant correctly misidentify that there has been no change). In addition, the response time with which each of these measures was recorded was obtained.

The four dependent measurements and response times were automatically recorded by the software and marked on the EEG. Specifically, response times were measured by the system from the presentation of the test stimulus until the recording of the participant’s mechanical response. From these recorded measurements, four more measures were calculated: accuracy, hit rate, false alarm rate, and Cowan’s K index for the estimated capacity of the VWM.

The accuracy, i.e., the measure of the overall task performance was calculated using Equation (1) by dividing the total number of hits and correct rejections by the total number of trials. Also, the hit rate Equation (2) and false alarm rate Equation (3) [27] were calculated to subsequently calculate the Cowan’s K estimated capacity.

(1) $$Accuracy=\frac{hits+correct\;rejections}{hits+correct\;rejections+misses+false\;alarms}$$

(2) $$\hat{h}=\frac{hits}{\left(hits+misses\right)}$$

(3) $$\hat{f}=\frac{false\;alarms}{\left(false\;alrms+correct\;rejections\right)}$$

Based on the conceptualization that the working memory consists of a series of limited slots in which the information is compartmentalized, if it is required to know how the number of available slots varies as a function of the condition (set size) and training session in a single probe CDT, the use of the VWM capacity estimator (Equation (4)) proposed by Cowan (denoted as *K*) is recommended, where N is the set size [28].

(4) $$K=N(\hat{h}-\hat{f})$$

Finally, for further analysis, the dependent variables considered were the accuracy, the response times of the hits and correct rejections, and the estimated capacity of the VWM (*K*).

### 2.3. Procedure

Participants performed the CDT while their EEG signals were simultaneously recorded. They were comfortably seated in a quiet, well-lit room, and were instructed to (a) look at the cross-shaped fixation point presented at the center of a 27-inch monitor (refresh rate: 60 Hz); (b) respond by pressing the right button (“n” key) with their right index finger when the test set was exactly the same as the stimulation set (square at the probed location did not change color compared to the memory stimulus set displayed) or to respond by pressing the left button (“s” key) with their left index finger when the stimulation set and the test set were different (square at the probed location did change color) (Figure 2B); (c) minimize blinking; and (d) respond as accurately as possible.

#### 2.3.1. EEG Recording

The electrical brain activity was recorded from the Fp1, Fp2, F3, F4, F7, F8, C3, C4,P3, P4, T3, T4, T5, T6, O1, O2, Fz, Cz, and Pz scalp locations, using non-invasive 6 mm Ag/AgCl gold cup electrodes (Natus, Middleton, WI, USA) topographically arranged according to the 10–20 international montage system and using a commercial silicone EEG cap. FPz served as the ground electrode, and all recording sites were referenced to CPz and re-referenced offline to the whole-scalp averaged reference. Inter-electrode impedance was maintained below 10 kΩ. The signals were acquired using an EEG amplifier (Natus XLTEK EEG32, Middleton, WI, USA) with a sampling frequency of 256 Hz, amplified at a band-pass of 0.5–70 Hz and attenuated at a band-stop of 60 Hz. Single-trial data were examined offline for averaging and posterior analyses.

#### 2.3.2. EEG Data Pre-Processing

The raw EEG signals were pre-processed in order to eliminate noise or artifacts that could affect the analysis. As a first step, a 4th order Butterworth cascaded digital band-pass filter with cutoff frequencies of 0.5–33 Hz was applied. This digital filter eliminated all those artifacts of frequencies lower or higher than those of the EEG rhythms of interest [29]. The artifacts that were embedded in the frequencies belonging to the EEG were also removed. To do this, a blind source separation (BSS) algorithm was used, which allowed the re-referenced signals to be separated into the different brain sources and isolate the non-brain sources for later elimination. Specifically, an algorithm of the Second Order Blind Inference (SOBI) family was used, named acsobiro, which is an algorithm that separates the signals, guaranteeing that they do not correlate at certain instants of time, which allows temporally correlated brain sources to be isolated [30].

**Figure 3:**
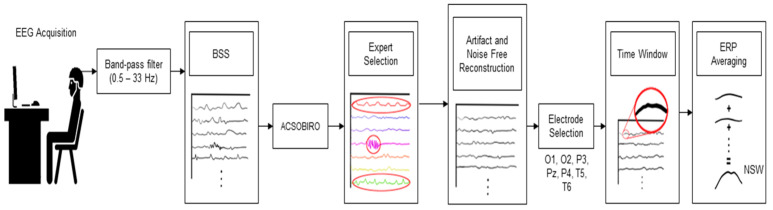
EEG data processing and ERP averaging. After being acquired, the data are band-pass filtered (0.5–33 Hz). Then, data were decomposed into their different components using BSS. Components related to noise or artifacts (circled in red in Expert Selection block) were removed by a trained expert. After reconstruction, the NSW-related time windows were obtained (see red circle in Time Window block).

Once the sources are estimated, the muscular, cardiac, and ocular artifacts are identified through visual inspection and excluded for clean EEG reconstruction, following correct guidelines to identify and eliminate them [31,32]. Visual inspection and artifact detection were performed by a trained expert blinded to groups. From this point on, only electrodes P3, P4, Pz, T5, T6, O1, and O2 were considered for subsequent NSW analysis, whereas this potential is mostly observable in the tempo-parietal and occipital regions [33,34,35]. The processing diagram can be seen in Figure 3.

#### 2.3.3. ERP Averaging

To obtain the ERP waveforms, the presentation of the memory set was taken as the initial time instant (t0). The epochs selected for ERP averaging corresponded to a time window spanning 100 ms before the memory array onset to 1050 ms after it. Fifty artifact-free trials were averaged for each set size in order to visualize the P300 component and the NSW. Before averaging, the 100 ms pre-stimulus period was used for the baseline correction. For each subject, individual average waveforms were obtained for the arrays with 2, 4, 6, and 8 squares during the 3 time points (pre-, mid-, and post-training). Then, these individual averages (nine per group, condition, and session) were used to obtain grand-mean waveforms for both the VWM group and the RT group. The subsequent analysis of the NSW features was carried out considering 2 time windows used in previous works that have studied certain properties of this component: one window of 480–900 ms post-stimulation [35] and another window of 500 ms post-stimulation at the end of the trial [36]. ERPs corresponding to all EEG recording channels are reported in the Supplementary Materials.

### 2.4. Data Analysis

The data obtained were analyzed in two ways: statistical analysis, to find significant differences between groups, conditions, and training sessions, and the ML analysis, to identify the NSW changes and the brain regions where they were detected.

#### 2.4.1. Statistical Analysis

The behavioral variables (mean accuracy, mean hit RT, mean correct rejection RT, and Cowan’s K values) were analyzed separately using repeated-measures (RM) ANOVAs. This approach has been used in previous studies to analyze similar behavioral and electrophysiological variables [35,36].

The electrophysiological data were analyzed using two 4 (Set Size: two, four, six, and eight) × 3 (Time: pre-, mid-, and post-training) × 7 (Electrode: P3, Pz, P4, T5, T6, O1, and O2) × 2 (Group: visual working memory video game training and reaction-time video game training group) RM-ANOVAs (one for each time window). This analysis aimed to explore general trends, effects, and interactions on the NSW mean voltage (mv). The ERP measure was calculated for each individual average waveform using two specified time windows that were consistent across all participants.

The analyses were performed using IBM SPSS Statistics 26 (IBM Corp., Armonk, NY, USA, released 2019). Greenhouse–Geisser corrections were applied to the degrees of freedom (df) as needed, and the corrected probabilities were reported. Additionally, post hoc tests were conducted with Bonferroni adjustments for multiple comparisons to explore any trends in the observed changes.

#### 2.4.2. Machine Learning Analysis

Due to the relatively small sample size of individual ERPs (9 per group, condition, and time point), we opted to perform the machine learning (ML) analysis on the EEG epochs used to obtain the NSW (450 per group, condition, and time point). This approach allowed us to significantly increase the amount of data available for training the ML models. The methodology of characterizing EEG epochs for decision tree (DT) training has been previously used to address classification problems related to learning and memory [37,38].

The models were trained to identify the features of NSW epochs that differentiate waveforms at different time points (pre-, mid-, and post-training) and to understand how these features differ between groups at a given time point. For the classification analysis, the following ERP measures were calculated for each EEG epoch: mean amplitude or mean voltage (mv), area under the curve (AUC), and coefficients for a 2nd degree polynomial (Q1, Q2, and Q3) fit for each time window. These measures were calculated for the same two specified time windows across all participants.

As previously mentioned, 49 epochs were derived from the 2-square set and 50 from the other sets, with each epoch consisting of the 7 selected EEG channels. Consequently, sets of 441 (49 epochs × 9 subjects) or 450 (50 epochs × 9 subjects) subsets of 35 features (7 channels × 5 features) representing each EEG epoch were obtained. In total, 48 such sets were created, one for each condition, training session, group, and time window (4 set sizes × 3 training sessions × 2 groups × 2 time windows).

These sets were used to train the algorithms and evaluate changes in the NSW epochs resulting from the training. The performance metric served as an indicator of changes in the participants’ waveforms: if the model performance improved, the difference between participants of different categories increased. Additionally, it is important to analyze the features that the algorithms identified as most decisive for the classification task.

##### Decision Trees for Classification

DTs are classifiers composed of decision nodes arranged in the form of a tree. This model learns a series of rules that optimally separate a set of data based on the features of the dataset [38]. DTs provide information about the most relevant features selected to achieve the optimal split of the data. They have shown average accuracy in detecting arousal and non-arousal states related to working memory consolidation during sleep [15] and pupillary response changes during VWM tasks [38].

By using several DTs, the model becomes a random forest (RF), which learns similarly to DTs, but in an ensemble manner. The RF model integrates the predictions of various DTs, each of which learns from random subsets of features [39].

To analyze how EEG epoch features changed as a function of video game training and group, we conducted a series of supervised bi-class classification analyses using the 48 feature sets in conjunction with DTs. The 48 sets were reorganized into two datasets: Dataset 1 and Dataset 2. Dataset 1 was used to observe how the features of the EEG signals differed as a function of training. Dataset 2 was used to observe how the features differed between groups at a given time instant. From this point forward, two specific strands were followed for each of the experiments. The 48 feature sets were organized as follows: In a first approach, for each of the 4 set sizes (2, 4, 6, and 8), 2 experimental windows (480–900 ms and 500–1200 ms) were tested on 2 groups (VWM, RT) for 3 time instances (pre-, mid-, and post-training). The second approach was very similar, but it only changed the 3 time instances by 3 time comparisons (pre vs. mid, pre vs. post, and mid vs. post).

##### Dataset 1: Training Differences

To determine whether the features of the NSW epochs were distinguishable across the three evaluation sessions (pre-, mid-, and post-training), we conducted a series of bi-class supervised classification analyses. These analyses were performed separately for each group and set size to observe specific changes related to the size of the stimulation set or the group.

The bi-class comparisons involved pairing the NSW features from different sessions. We compared the following: pre-session vs. mid-session, pre-session vs. post-session, and mid-session vs. post-session.

This approach required reorganizing the original 48 primary sets to fit the bi-class comparison format. The new sets had the following structure: 2 time windows (480–900 ms and 500–1200 ms) × 4 set sizes [2,4,6,8] × 3 time comparisons [pre- vs. mid-, pre- vs. post-, or mid- vs. post-] × 2 groups [VWM, RT]. Each set consisted of 441 or 450 instances (depending on the set size), with each instance representing an EEG epoch from one of the 9 participants.

Each instance had 36 attributes, which included 7 electrodes (O1, O2, P3, P4, Pz, T5, and T6), 5 features (maximum voltage, area under the curve, quadratic coefficient, linear coefficient, and constant), and 1 class label (indicating the session comparison: pre- vs. mid-, pre- vs. post-, or mid- vs. post-).

The classification was carried out using DT classifiers. For each classification task, 80% of the data were used for training/validation, and the remaining 20% were used to test the models. The hyperparameters for the DT models, such as the splitting criterion, maximum depth, minimum number of samples per leaf node, and the number of features considered for the best split, were optimized using a grid-search approach. Each classification task (48 in total) had its hyperparameters optimized. The models were validated using 5-fold cross-validation (k = 5).

The F1 score, which is a weighted average of precision and recall, was chosen as the performance metric [40]. All analyses were conducted using the scikit-learn library [41] in Python (v.3.26.0).

##### Dataset 2: Group Differences

We conducted supervised bi-class classification analyses to assess whether EEG epoch features differed between groups at specific time points, such as pre-, mid-, or post-training, and across various set sizes. Data from the original 48 main sets were reorganized into 24 sets combining both the VWM and RT groups. Each new set included 4 set sizes (2, 4, 6, 8), 3 time points, and 2 time windows (480–900 ms and 500–1200 ms). Each set contained 882 or 900 instances, depending on set size, representing EEG epochs from 9 participants per group.

Each instance comprised 36 attributes: 7 electrodes (O1, O2, P3, P4, Pz, T5, and T6), 5 features (maximum voltage, area under the curve, quadratic coefficient, linear coefficient, and constant), and 1 class label indicating group membership (memory video game training or active control).

For analysis, we utilized decision tree (DT) classifiers, with 80% of the data used for training/validation and 20% for testing. Hyperparameters were optimized via grid search, and models were validated using 5-fold cross-validation (k = 5). The F1 score, a weighted average of precision and recall, served as the performance metric.

## 3. Results

This section presents the results obtained in both domains, the behavioral changes reflected in the change detection task and the electrophysiological changes observed in the NSW due to the size of the memory set, and the cognitive training program completed with the video games. These results seek to demonstrate whether there were benefits in accuracy, RTs, and estimated VWM capacity, as well as changes in NSW that underlie the behavioral benefits.

### 3.1. Change Detection Task Analysis

Exploring the differences in performance between the groups due to the video game training was the main interest of the change detection task statistical analyses. The RM-ANOVAs were performed separately on mean accuracy, mean hit RT, mean correct rejection RT, and the Cowan’s K values.

#### 3.1.1. Accuracy

To begin with, we found a significant main effect of the set size [F(4, 64) = 136.98, *p* < 0.001, $\eta_{p}^{2}$ = 0.89] and of the time [F(2, 32) = 4.40, *p* = 0.020, $\eta_{p}^{2}$ = 0.21]. We found greater accuracy values (ratio of correct responses) on smaller set sizes and at the mid-training evaluation session, respectively (Table 1). A significant time × set size interaction was also observed [F(8, 128) = 4.02, *p* = 0.001, $\eta_{p}^{2}$ = 0.20].

To examine these interactions and examine whether there were changes in this measure as a function of training or between groups, a series of pairwise comparisons (*t*-tests) with a Bonferroni correction were performed. It was found that the accuracy values were higher in the mid-training session when compared to the other two sessions (t(16) = −2.7, *p* = 0.048, t(16) = −2.67, *p* = 0.049). Both groups presented an increase in their accuracy for the 2-square set from the mid- to the post-session (t(16) = −3.98, *p* = 0.003). In turn, both groups decreased their accuracy values from the mid- to the post-session for the eight-square set (t(16) = −3.68, *p* = 0.006).

#### 3.1.2. Mean Hit Reaction Time

Significant main effects of set size [F(4, 64) = 22.10, *p* < 0.001, $\eta_{p}^{2}$ = 0.58] and time [F(2, 32) = 4.95, *p* = 0.013, $\eta_{p}^{2}$ = 0.23] were also found with a lower mean hit RT on smaller set sizes and at the post-training evaluation session. Comparisons showed that the lowest hit RT values were presented by both groups for the two-square set when compared to the four-, six-, and eight-square sets (t(16) = −4.65, *p* = 0.001, t(16) = −7.95, *p* < 0.001, t(16) = −6.03, *p* < 0.001, respectively). In addition, the lowest values were also observed in the post-session compared to the mid one (t(16) = −3.4, *p* = 0.010) (Table 2). Both groups presented minor RTs at the post-training session.

#### 3.1.3. Mean Correct Rejection Time

Results for the correct rejection RT analysis showed a significant main effect of set size [F(4, 64) = 26.50, *p* < 0.001, $\eta_{p}^{2}$ = 0.62] and time [F(2, 32) = 10.85, *p* < 0.001, $\eta_{p}^{2}$ = 0.40], with lower correct rejection mean RT on smaller set sizes and at mid- and post-training sessions, respectively. Also, a significant set size × group interaction was found, F(4, 64) = 2.90, *p* = 0.029, $\eta_{p}^{2}$ = 0.15 (Table 3). The VWM group had lower mean RTs on the two-square set size and similar RTs in the trials with the four- and six-square arrays and with the six- and eight-square array trials (t(16) = −2.90, *p* = 0.097, t(16) = −1.35, *p* = 1.00, respectively). In comparison, the RT group presented similar mean RT on the two- and four-square array trials and the four- and eight-square trials (t(16) = −1.96, *p* = 0.63, t(16) = −1.97, *p* = 0.66, respectively).

#### 3.1.4. Cowan’s K Values

Regarding the Cowan’s K values (VWM capacity estimate), significant main effects of set size [F (4, 64) = 16.32, *p* < 0.001, $\eta_{p}^{2}$ = 0.50] and time [F(2, 32) = 6.29, *p* = 0.005, $\eta_{p}^{2}$ = 0.28] emerged with greater values at the mid-training evaluation session for the greater set sizes (four-, six-, and eight-square). A significant time × set size interaction was found [F(8, 128) = 4.35, *p* < 0.001, $\eta_{p}^{2}$ = 0.21] (Table 4).

The estimated capacity values were higher in the mid-session when compared to those of the pre-session (t(16) = −2.87, *p* = 0.033), but lower when compared to those of the post-session (t(16) = −3.49, *p* = 0.009).

Both groups presented an increase in their capacity from the mid-session to the post one for the two-square set (t(16) = −0.41 *p* = 0.003). At the same time, a decrease in this value was observed when comparing the mid-session with the post one for the eight-square set (t(16) = 3.68 *p* = 0.006). This coincides with the increase in accuracy values when comparing the same conditions. It should be noted that this value reflects the behavior observed in the accuracy values. Moreover, it was observed that the values during the mid-session where the value of the estimated capacity are significantly lower for the two-square set compared to the four-, six-, and eight-square sets (t(16) = −13.51, *p* < 0.001, t(16) = −6.90, *p* < 0.001, t(16) = −5.06, *p* = 0.001, respectively).

### 3.2. Negative Slow Wave Analysis

In order to identify the relationship between the memory set size and the NSW component, two separate RM-ANOVAs, one for each window, with the set size, training session, and electrode site as the within-subject factors and the group as a between-subject factor, were performed on the NSW mean voltage calculated in the 480 ms to 900 ms and 500 ms to 1200 ms time-windows, since these are the periods where the most notable differences between grand-mean waveforms were observed by previous works [35,36]. The electrode sites considered in this analysis were P3, P4, Pz, T5, T6, O1, and O2.

For both windows, a main effect of set size was found [F(3, 48) = 22.45, *p* < 0.001, $\eta_{p}^{2}=0.58$, F(3, 48) = 15.18, *p* < 0.001, $\eta_{p}^{2}=0.49$, respectively] with greater negative mean voltage for the larger set sizes in both groups.

However, no significant training effect was found for any window [F(2, 32) = 0.87, *p* > 0.05, $\eta_{p}^{2}=0.00$, F(2, 32) = 0.042, *p* > 0.05, $\eta_{p}^{2}=0.00$, respectively]. Nor was any significant interaction found between training × set size [F(6, 96) = 0.27, *p* > 0.05, $\eta_{p}^{2}=0.08$, F(6, 96) = 1.68, *p* > 0.05, $\eta_{p}^{2}=0.09$, respectively].

**Figure 4:**
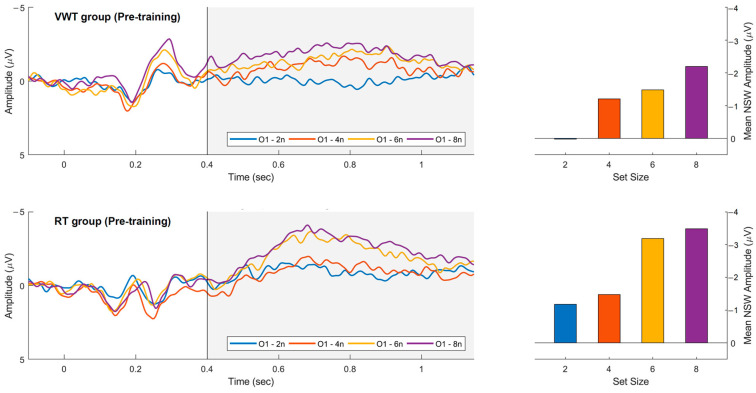
Relation between the Negative Slow Wave (NSW) amplitude at the O1 electrode site and the memory set array size for the pre-training session in both groups. Significant larger negative amplitudes were observed for the larger-sized sets in both groups, in addition to a step-wise change in potential amplitude as a function of set size (480–900 ms). The shaded area corresponds to the NSW (400 ms 1200 ms). The dark vertical line represents the onset of the visual stimulus.

Figure 4 shows the behavior of NSW in the left occipital region as a function of the set size in the pre-training session. The mean voltage was calculated over the 480 ms to 900 ms window. It is possible to observe a step-wise behavior in the voltage, which increases as a function of the number of squares.

This allows us to infer that the amplitude of the NSW component increases as the set size of the memory set also increases. Nonetheless, the changes in this step-wise pattern were not significant for the mid- and post-training sessions. The mean voltages of the NSW during this time window are observable in Table 5.

Although no significant training effects were found, it was found that there are significant training differences in electrode O1 for the two-square task in the VWM group. This is observed when comparing the mean voltage of the pre-session with the mid-session (t(16) = −2.32, *p* < 0.05) and that of the mid-session with the post-session (t(16) = −2.34, *p* < 0.05) for the 500 ms to 1200 ms time window. However, this is a very particular result that only occurs in one set size, so it is difficult to generalize.

### 3.3. Classification Analysis

The results of this section are presented in two subsections corresponding to each experiment performed.

#### 3.3.1. Classification Results: Dataset 1

The F1 scores obtained by the DT when comparing the NSW epochs’ features of the VWM group, obtained from the 480–900 ms window, are shown in Table 6.

The scores highlighted in bold are the highest scores achieved by the DT for each time instant comparison. The highest value is reached when comparing the pre- and post- sessions of the VWM group for the set of two squares (0.73), whereas the highest score reached by the RT group appears while comparing the mid- and post- sessions for the four-square set (0.69). The other highlighted values show that the set of features allows for the differentiating of the waveforms at different time instants, to a greater or lesser extent, whereas the lower values indicate that, for that comparison, the features are less differentiable; therefore, it is assumed that there are no noticeable changes between these EEG epochs.

The pre-, mid-column shows that the VWM group achieves higher values than those of the RT group, except for the four-square set. This seems to indicate that the features of the VWM group vary more from pre- to mid-session than those of the RT group.

**Figure 5:**
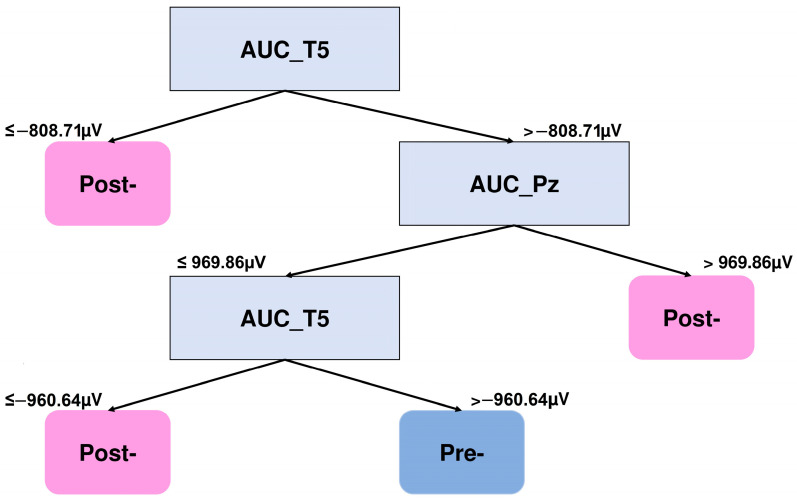
DT graph to distinguish the NSW features of the VWM group. The area under the curve of electrode T5 (AUC_T5) and the area under the curve of electrode Pz (AUC_Pz) are the features that better distinguish between the pre- and post-training 2-square NSW epochs.

In order to visualize which features the DTs consider to perform the best separation of the NSW features, the graphical representations of the algorithm that produced the highest classification results for each group were obtained. The DT corresponding to the highest result obtained for the VWM group (0.73) is presented in Figure 5. It is observed that the features that allow for the distinction between the pre- and post-training NSW epochs are the area under the curve of electrode T5 (AUC_T5) and the area under the curve of electrode Pz (AUC_Pz). Specifically, the tree indicates that the value of the AUC of the left temporal region changes as a function of training. That is, AUC values less than −808.71 μV are related to the later session, and, hence, the amplitude values become more negative in the post-training session. On the contrary, if the AUC of the T5 electrode is greater than −960.64 μV and the AUC of the electrode Pz is less than 969.86 μV, the epochs are related to the pre-training session.

**Figure 6:**
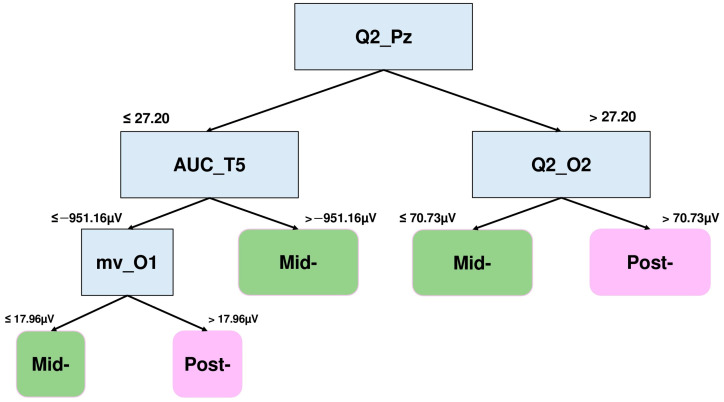
DT graph to distinguish the NSW features of the RT group. The lineal coefficient of the 2nd degree polynomial fit of electrode Pz (Q2_Pz), the area under the curve of electrode T5 (AUC_T5), the linear coefficient of electrode O2 (Q2_O2), and the mean voltage of electrode O1 (mv_O1) are the features that better distinguish between the mid- and post-training 4-square NSW epochs.

The DT graph for the highest score reached for the RT group can be seen in Figure 6.

In this case, the linear coefficient of the second degree polynomial fit of electrode Pz (Q2_Pz), the area under the curve of electrode T5 (AUC_T5), the linear coefficient of electrode O2 (Q2_O2), and the mean voltage of electrode O1 (mv_O1) are the features that better distinguish the mid- and post-training of the RT group.

Specifically, Q2_Pz values greater than 27.20 in conjunction with Q2_O2 values less than or equal to 70.73 μV are related to the mid-session, while those greater than 70.73 μV characterize the later session. Moreover, Q2_Pz values lower than 27.20 in conjunction with AUC_T5 values greater than −951.16 μV occur in the mid-session. On the contrary and in conjunction with mv_O1 values greater than 17.96 μV, the features correspond to the later session.

Congruent with what was seen for the VWM group, the RT group also presents a more negative amplitude in the left temporal region for their post-training session but a more positive mean voltage in the left occipital region.

The F1 scores obtained by DT when comparing the features considering the time window from 500 ms to 1200 are shown in Table 7.

The highest F1 score achieved for the VWM group appears when comparing the pre- to the post-training session in the two-square condition (0.72). This result is consistent with that obtained for the same conditions when considering the window from 480 ms to 900 ms. Meanwhile, the RT group presents the most differentiable features when comparing the mid-session with the post one in the two-square condition (0.70). Similarly, considering this wider time window, it is possible to observe that the F1 scores of the VWM group are higher than those of the RT group when comparing the pre- and mid-training sessions for all conditions.

**Figure 7:**
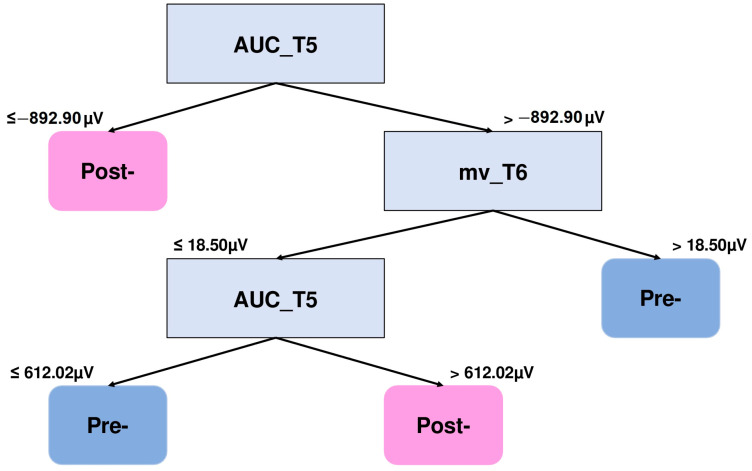
DT graph to distinguish NSW features of the VWM group (500–1200 ms). The area under the curve of electrode T5 (AUC_T5) and the mean voltage of electrode T6 (mv_T6) are the features that better distinguish between the pre- and post-training two-square NSW epochs.

Figure 7 shows the DT corresponding to the comparison with the highest F1 score of VWM group for the 500 ms to 1200 ms window. It is observed that the features considered for this classification are AUC_T5 and the mean voltage of electrode T6 (mv_T6). Again, a more negative voltage in the left temporal region is related to the post-training session, whereas a less negative voltage in that region in conjunction with a mostly positive average voltage value in the right temporal region describes the previous session. Conversely, a less positive mean voltage at T6 in conjunction with a greater AUC_T5 value than 612.02 μV indicates post-session features.

**Figure 8:**
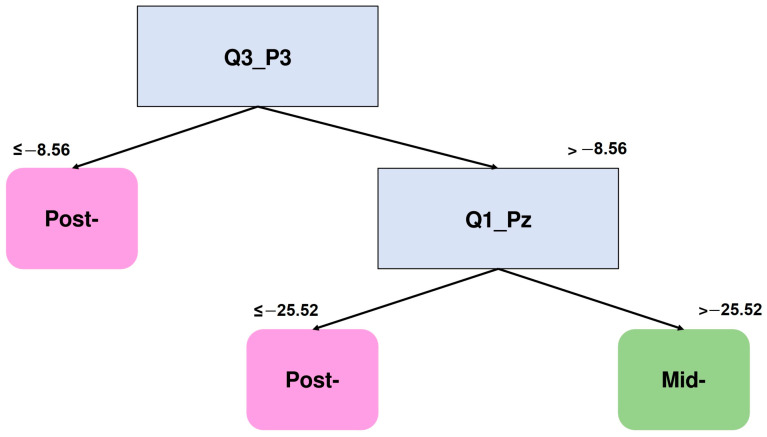
DT graph to distinguish NSW features of the RT group (500–1200 ms). The 2nd degree polynomial constant coefficient of electrode P3 (Q3_P3) and the 2nd degree polynomial quadratic coefficient of electrode Pz (Q1_Pz) are the features that better distinguish between the post- and mid-training 2-square NSW epochs.

Finally, Figure 8 shows the DT corresponding to the comparison between the mid- and post- training sessions of the two-square condition of the RT group for the time window from 500 ms to 1200 ms. According to this tree, the constant of the polynomial approximation at electrode P3 (Q3_P3) and the quadratic coefficient of this approximation at electrode Pz (Q1_Pz) are the features considered to make this distinction.

Specifically, mostly negative values of Q3_P3 describe the post-training session. Conversely, when Q3_P3 values greater than −8.56 are present, Q1_Pz is considered, where values less than −25.52 are related to the post-session. The quadratic coefficient indicates that the waveforms tend to be more convex at the post-session, while values of this coefficient of more than −8.56 are related to the mid-training NSW epochs; that is, the waveforms tend to be more convex.

#### 3.3.2. Classification Results: Dataset 2

The F1 scores obtained by the DT when comparing the NSW epochs features of the groups obtained from the 480 ms to 900 ms window are shown in Table 8. The scores highlighted in bold are the highest scores achieved by the model for each group comparison.

**Figure 9:**
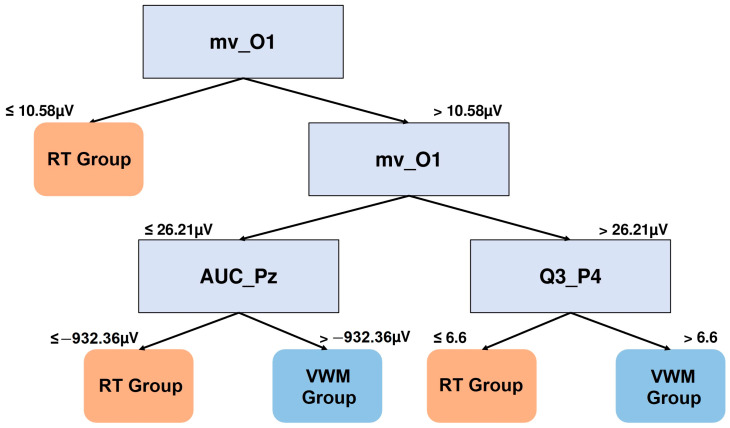
DT graph to distinguish NSW features of the groups in the mid-training session (480–900 ms). The mean voltage of electrode O1 (mv_O1), the area under the curve of electrode Pz (AUC_Pz), and the constant of the polynomial fit of electrode P4 (Q3_P4) are the features that better distinguish between the groups for the mid-training 8-square NSW epochs.

The DT shows that the mid-session features are mostly distinguishable for the eight-square condition (0.71). This tree is shown in Figure 9.

Specifically, the VWM group is characterized by presenting the mean voltage values in the range of 10.58 μV and 26.21 μV in the left occipital region (mv_O1), together with area under the curve values at the Pz electrode (AUC_Pz) greater than −932.36 μV.

For the 500 ms to 1200 ms window, the results are shown in Table 9. The results reveal that the groups are mostly different when comparing the groups at the mid-session (1 month of training later) for the two-square condition (0.71). Additionally, the DT shows that the F1 scores obtained for all conditions in the pre-session are lower than those in the mid-session. This implies that for this time instant, the features of the groups are largely indistinguishable.

**Figure 10:**
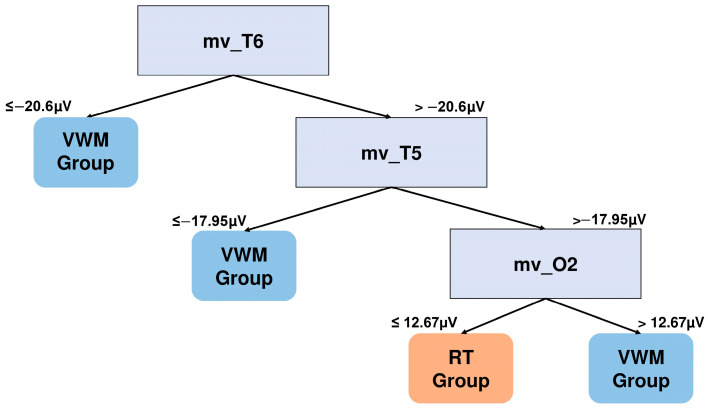
DT graph to distinguish NSW features of the groups in the mid-training session (500–1200 ms). The mean voltage of electrode T6 (mv_T6) and the mean voltage of electrode O2 are the features that better distinguish between the groups for the mid-training 2-square NSW epochs.

Figure 10 shows the tree corresponding to the comparative with the highest F1 score (mid-session, two-square condition).

The VWM group is characterized by a more negative mean voltage in the temporal region, while the RT group presents more positive values in this region together with more negative mean voltages in the right occipital region.

## 4. Discussion

In this study, we examine the changes in behavior in a change detection task and in the NSW component in a complementary manner, which emerge in the visual information retention period of this task, resulting from two video game training conditions. The obtained results show that the designed change detection task provides results in line with previous works that have applied this paradigm to the study of VWM, such as higher accuracy values for smaller set sizes (Table 1) and higher Cowan estimator values for the larger sized memory sets [5,35].

### 4.1. Behavioral Correlates of Simple Video Game Training

The accuracy in the change detection task obtained differs for each condition; specifically, greater accuracy was observed in smaller set sizes, and this behavior is congruent with previous studies where accuracy was studied as a function of the set size of the change detection task [5,13].

Regarding the effect of the proposed training on the accuracy values, we found a significant increase in the smallest set size condition (two-square) when comparing the mid- with the post-training session. In addition, we found a significant decrease in the accuracy value in the eight-square condition. These changes were observed for both groups. Moreover, the highest accuracy values are observed after one month of training. Changes related to the training were observed for both groups, which differs from the initial hypothesis; this means that training with the video game played by the RT group also led to important changes. However, the question also arises as to whether the observed benefits are specific to training with simple video games or are due to some test–retest effect of the CDT task.

In contrast to our findings, in previous studies in which an action first-person shooter video game [5] was used for VWM capacity training, significant benefits were reported in the accuracy of four-, five-, and six-square set sizes. Similarly, it has been shown that expert players in ARS video games reach higher accuracy values than non-experienced players in a change detection task of one, two, and four squares [13]. Both studies make use of action video games for training and attribute the increases in accuracy to aspects of the genre, such as the participants responding to a stimulus under pressure, high cognitive demand, divided attention, and changes between periods of concentration and divided attention. The lack of changes in the accuracy value in bigger set sizes (four-, six- and eight-square) observed in this work can be attributed to the genres of video games used for training (i.e., 2D puzzle simple games) and also to the fact that in our experiment, participants were not obliged to respond under pressure, since there was no time limit in the test set phase. However, a similar lack of effect of training on the accuracy values of this task (two, four, six, and eight squares) has been previously reported [26]. When using a simple attentional task adapted as a mobile app to train the VWM, no significant effects were found on the accuracy, but rather on the response time in the change detection task.

We found that both groups take less time to respond (hit and correct rejection) to the two-square set in the post-training session. It was for the correct rejection RT where we found an interaction between the set size and the group. It seems that this interaction is mediated by the apparent difference in this value in the two-square, pre-session condition between the two groups; however, this difference is not significant (t(16) = −1.23, *p* = 0.23). The behavior of the groups is quite similar for the other conditions (Table 3). As stated before [42,43], the quality of the information encoded in the VWM can be measured through the RTs. In other words, dealing with visual information in an optimal way allows for shorter retrieval times [44]. Thus, the decrease in RT in the simplest condition of the change detection task (two-square) could be considered as a benefit of our training with simple video games in the VWM group. Since this benefit is not applicable to the RT group, it can be directly attributed to the training with the simple video game designed for memory.

The Cowan estimator changed over time in both groups. The mean Cowan estimator values for the VWM group were K = 3.06, 3.77, and 2.56 (for the pre-, mid-, and post-training sessions, respectively) and 3.16, 3.27, and 2.16 for the RT group. These results allow us to infer that the participants of both groups improved the estimated capacity of their VWM while comparing the pre- and mid-training capacity values. Congruent with the accuracy values, the participants presented an increase in the estimated capacity for the two-square array in the post-training session. Moreover, it was in the session after one month of training where a progressive increase in this value as a function of set size was noted.

Also, both groups showed a decrement of this value, even lower than the pre-training values, for the post-training session for the eight-square condition (see Table 4). This behavior, where the VWM measures decrease considerably in the post-training session, has not been reported in previous works that use video game training to train the VWM [5,26]. This decrease could be attributed to some confounding variable, which cannot be measured with the methodology proposed in this work; such is the case of the motivation, expectation, or engagement that the participants had before and after their training. It is possible that the participants experienced fatigue, low motivation, or interest in this condition of the task. It is worth remembering that for the post-training evaluation, the participants came from training with the highest difficulty of the video games, which increased progressively as a function of time. However, these assertions cannot be made without the support of an exit survey that would allow us to know these variables. Previous works that have not found this behavior have made use of adaptive training where the difficulty of the training is modified according to the demands of the participant [26].

In sum, contrary to our hypothesis, both groups presented changes and benefits in the dependent measures considered, including increased accuracy, shorter response times for the same condition, and an increase in the estimated capacity of the VWM for the two-square condition. However, these benefits are not directly attributable to training with simple video games. Except for the VWM group, the RT correct rejection was lower for the two-square condition than for all other conditions, whereas the RT group exhibited similar RTs regardless of the condition.

### 4.2. Neural Correlates of Simple Video Game Training

Current results show the significant sensitivity of ERP amplitudes to the number of elements in the memory set, with more negative amplitudes for larger set sizes and similar amplitude values for set sizes that exceed VWM capacity, specifically for sets of six and eight squares. These findings are consistent with what has been reported in the literature about the NSW component as a neural correlate of the change detection task [22,33,35,45]. We found that this relationship tends to be less pronounced as the training sessions progress and was not significant in the mid- and post-training session evaluations. The NSW is more robust at posterior EEG electrode sites (parietal, temporal, and occipital) [46].

The NSW analysis did not reveal significant effects of video game training on the average voltage of this potential, with only a couple of significant differences observed at electrode O1 for the two-square condition in the VWM group. Although these results could imply that training with simple video games was not effective in enhancing VWM capacity, we chose to analyze the average voltage of the component along with four additional features: the area under the curve (to assess the energy of the waveforms) and the terms of the second-degree polynomial approximation. This approach was used because the NSW resembles a quadratic function, whose apex is related to the retention period of the change detection task, as well as the amplitude and curvature of the potential. In addition, considering the small sample size, it was decided to use DTs on EEG epoch features instead of analyzing the grand averages of the NSW. In this way, it was possible to increase our sample in order to explore the possibility that there might be changes that machine learning could cause.

### 4.3. Decision Trees Provide Insight on Cognitive Benefits

The obtained features were used to train ML models to determine whether the sets were distinguishable based on time instant or group, i.e., to analyze whether there were changes in the NSW epochs. The results from these models would indicate the degree of distinction, helping us understand if and how the features of the potential changed. Therefore, we characterized the EEG epochs to increase the amount of data available for training the machine learning models. As previously discussed, characterizing EEG waveforms for training ML models is a methodology that has been used to classify various brain states, such as cognitive load and learning, among others [15,16,37]. However, in this study, we chose to characterize the epochs used for averaging and obtaining ERPs. This allowed us to leverage ML models that specialize in identifying characteristic patterns in large datasets, which may not be immediately apparent.

The ML algorithms allowed distinctions to be made between the different sets of features obtained from the NSW epochs.

DTs revealed that the highest F1 scores achieved for the VWM group considering the 480 ms to 900 ms time window occur when comparing the NSW epoch features of the pre- and post-session NSW epochs for the two-square condition. Similarly, DTs found the highest F1 score for this comparison when considering the 500 ms to 1200 ms window.

The decision trees (see Figure 6 and Figure 7) show that this group presents more negative AUC_T5 values in the session after two months of training. Such a change can be related to the training with the simple memory video game, as it is not presented for the RT group. In addition, the post-training session is also related to more negative AUC_Pz and mv_T6 values compared to the pre-training session values.

The topographical distribution of the NSW has been described in terms of the different cognitive processes it might reflect. Recently, Zickerick and colleagues examined the fronto-central slow wave as an index of WM maintenance [47], which is thought to reflect processes of cognitive control associated with the maintenance of task-relevant information [46,48], but they additionally investigated the posterior slow wave which has been associated with cognitive operations following target identification [49,50]. According to these authors, an increase in a more centrally distributed component may indicate improved WM maintenance and rehearsal activity.

In the light of our findings, we could argue that the observed increased negativity in temporoparietal regions, after training in the memory group, may indicate improved WM maintenance and rehearsal activity. Functional magnetic resonance imaging studies have proved that cortical activation in WM tasks is not limited to the prefrontal cortex [51,52,53], but that co-activated regions have been found consistently in the inferior temporal cortex and the posterior parietal cortex. The inferior temporal cortex shows greater sensitivity to the identity and features of a stimulus, such as shape or color, independent of the stimulus location [54,55], whereas a location-specific signal in the posterior parietal cortex has shown the strong retinotopic mapping of a remembered target location [56]. According to [57], the left IPS plays an important role in the phonological storage during VWM; they suggest that stimuli appear to be recoded and maintained by verbal rehearsal in a phonological short-term store in virtually similar regions, regardless of if they are auditory or visual. In fact, the left lateralized effect, observed over the T5 scalp region, could be associated with a verbal active rehearsal of visual and spatial information, meaning it is not only related to posterior visual areas activation. As for the RT group, it is possible to observe more negative AUC_T5 values for the later session; however, they are in conjunction with mostly positive mv_O1 values and for the four-square condition.

On the other hand, for both time windows, the DTs show that the F1 scores obtained when comparing the pre- and intermediate sessions of VWM group for all set sizes are higher contrasted with those of the RT group. Thus, it can be inferred that the features of the VWM group are more distinguishable between these time instants than those of the RT group. This may indicate that the simple memory video game was able to present greater changes in the EEG waveforms, unlike the video game used for the training of the RT group, after 1 month of training.

Also, the DTs show that the greatest changes for the RT group are observed when comparing the mid- and post-training sessions, specifically for the two- and four-square conditions, i.e., after 2 months of training. Among these changes, more negative AUC_T5 values are observed in the post-session, which coincides with what was observed for the VWM group.

Both groups show a change in AUC_T5 for the post session; however, for the RT group, this change is for a larger set size and occurs after 1 month-long training. In addition, the VWM group also shows more negative values in the right temporal region (T6), while the RT group does not.

With respect to the groups, the DTs show that the F1 scores of the previous session are lower than those of the mid-session. This lets us know that the features of the groups before training with the video games are practically indistinguishable, which allows us to attribute the changes in the other sessions to the training.

Finally, the DTs show that the groups differ mostly in the intermediate session for the two- and two-square conditions. Changes in the two-square condition again imply more negative values in the temporal region compared to the RT group, while the RT group also presents more positive values in the right occipital region for the same condition.

### 4.4. Video Game Design Factors

Contrary to what has been reported in the literature, in which commercial action [5,14,58] or adventure [24] video games are utilized for VWM training, we decided to design our own platform as a hybrid between a commercial video game and a cognitive trainer, since this allowed us to have the freedom to modify various aspects of the game, such as its visual components, its difficulty, or adding competitive factors such as a scoring table that lists the best scores obtained by the participants. In addition, it allowed us to control the game time of the participants since they could do the training from home at the time they preferred to do it. This was in order for the subject to take his training in the most enjoyable way possible, thus avoiding possible negative effects related to the participants seeing their training as a tedious task. Moreover, factors such as fun, motivation to play, constant difficulty changes, and rewards have been identified as factors that directly influence the video game training of cognitive abilities [59,60,61]. The use of hybrid platforms has become popular because of its positive results in cognitive training [62]. The changes related to the Cowan estimator show an improvement for the mid-training session, while they tend to return to baseline for the post-training session. This may be due to the fact that, in general, the participants completed fewer levels in the second month of training compared to the first one. The levels of the second training period were more difficult, in addition to the fact that the difficulty remained monotonously hard; that is, the game became more complex than it already was, which could be an important factor for some participants to play less time. Perhaps adding dynamic difficulty changes, that is, varying the difficulty non-progressively so that the game does not remain simple or complex for a long period of time, should be considered. This could help maintain the participants’ interest in continuing their training. The exploration of this relationship in the present work is limited, since there is no exhaustive information from the participants that describes how they felt when performing the training.

### 4.5. Limitations

While the present study provides a novel exploration of the benefits of training in working memory by combining ERP measures and machine learning, it has limitations. Research has shown that participants tend to benefit more from cognitive training that adapts its difficulty based on their individual performance. In other words, the training difficulty should change in response to how well participants are performing. In this study, however, we used progressive changes in difficulty, where the difficulty varied as a function of time rather than participant performance. To this, we can attribute the lack of cognitive benefits in the post-training session, as participants probably experienced a lack of motivation to perform their training or were faced with a very complex task to solve. The change in difficulty could be modulated through performance data recorded in the simple video game or through a cognitive estimation algorithm, the latter of which would involve parallel EEG monitoring in training, which could result in a negative effect on the participant. However, in order to determine the lack of benefit, it would be necessary to implement exit surveys that would provide us with information on the participant’s perception of the training video game, the change detection task, and video game training in general. Also, the transfer effects of the proposed training are needed. Participants were trained with a memory video game and tested on a change detection task. However, it is necessary to assess whether memory benefits in complex tasks that require other cognitive domains, such as attention, are closer to participants’ everyday tasks. Likewise, a non-immediate post-training evaluation session is needed at least three months after training in order to monitor changes and whether they are maintained as a long-term benefit. We believe that the major limitations of our work are in the sample size and in the blinding used in the experimental design. The relatively small sample can explain the lack of robust behavioral (accuracy, RT, and VWM estimated capacity) and electrophysiological (NSW) results obtained. We tried to solve this problem by performing ML analysis, multiplying the NSW data of each participant, which allowed us to find more robust results about the change in NSW in the temporoparietal regions. In turn, the experimental design was such that participants knew the experimental condition, the other control condition, and which one they belonged to from the beginning of the study. The lack of blinding may attenuate our results, as participants’ behavior could have been biased, even unconsciously. Therefore, it would be necessary to thoroughly examine the effect of this part of the experimental design in order to increase the robustness of our results.

The present study uncovered minor changes in the dependent CDT. However, the observation of these benefits in both the VWM group and the RT group raises the question of a potential test–retest effect related to the change detection task, a phenomenon previously documented in cognitive research [5,21,63]. This test–retest effect could lead to improved performance due to familiarity rather than the training intervention itself. To further investigate this possibility, incorporating a test–retest reliability analysis [5] could serve as a valuable tool to discern the extent to which the observed enhancements are influenced by this effect. The need for the careful consideration of experimental design, control tasks, and group assignment within the context of training paradigms and the test–retest effect should be emphasized. Addressing these concerns and utilizing robust methodologies is crucial in drawing meaningful conclusions from cognitive training studies. However, it was found that the VWM group became faster at responding correctly to a correct rejection of the CDT, a behavior that was not present in the RT group. In addition, the changes in brain electrical activity in the temporoparietal region in the same group could indicate that the training with the memory video game brought behavioral benefits and changes in the group that trained with it.

Finally, our study lacks additional neuropsychological measures of working memory capacity or other executive functions, such as attentional control, which would allow a more precise characterization of our sample and differential analysis of the effect of training in relation to another parameter. While the participants in the present study were young cognitively normal individuals, we believe that the training model could be applied to explore improvement capacity in patients who have some deficits in WM capacity or other cognitive deficits that correlate with WM capacity.

### 4.6. Practical Implications

The findings of this study underline the potential of video game-based training to improve cognitive abilities, particularly visual working memory (VWM). These results suggest the following practical applications in several areas:

1. Cognitive Enhancement and Rehabilitation: Video game training has been shown to enhance VWM and attentional control, as evidenced by improvements in performance after targeted interventions [5]. These findings suggest that video games could be used as a cost-effective tool in rehabilitation programs for individuals with cognitive impairments, such as those recovering from brain injuries or dealing with age-related memory decline.
2. Educational Applications: The cognitive benefits observed from video game training, such as enhanced memory retention and multitasking skills, align with the needs of educational settings [62]. Incorporating video game-based activities into the curriculum could help students develop these crucial skills in an engaging and interactive way.
3. Professional Skill Development: Fields that demand quick decision-making and sustained attention, such as healthcare, aviation, and military operations, could benefit from integrating video game training into their training programs. Research has shown that video games provide environments that mimic real-world cognitive demands, fostering improvements in performance under pressure [5].
4. Therapeutic Gaming: Video games designed to target specific cognitive skills could serve as therapeutic tools for conditions like ADHD or mild cognitive impairment. The engaging nature of video games ensures consistent participation and motivation, which are key factors in the success of therapeutic interventions [62].

## 5. Conclusions

The present pilot study provides evidence to demonstrate that training with a simple video game, a hybrid between a commercial video game and a cognitive trainer, generates changes in non-professional gamer subjects who trained during a brief 2-month period. We found changes that could suggest improvements in accuracy, the response time with which encoded visual information is retrieved, and estimated visual working memory capacity, specifically in the simplest condition of a change detection task (two-square). From another perspective, the fun factor seems to play an important role in maintaining the attention and motivation of the subjects who train. As future work, it has been proposed to evaluate the subjects with a battery of cognitive tasks that provide information on the cognitive skills of interest and others that may be being trained in parallel, or to identify if the favorable changes seen in a certain domain imply negative changes in other domains. Regarding the design of video games, it is proposed to focus on the fun of the subject in order to match the characteristics of commercial video games and also to offer a cognitive benefit through the aforementioned factors, as long as the subject has the motivation to continue with the training and also finds it challenging and entertaining. In addition, it is important to consider adaptive changes in difficulty, which maintain the interest and motivation of the subjects to continue their training. Altogether, the video game training of cognitive abilities is an area of interest that deserves future attention from both the neuroscience and machine learning perspectives, specifically in young adult populations, where the first signs of cognitive decline have been shown to occur. From the neuroscience perspective, we must seek to understand the neural mechanisms underlying the different cognitive domains and how these can benefit from attractive, fun training that does not represent a tedious task, such as video games. Machine learning tools help us to evaluate such training and to identify changes at the neuronal level through the classification or association of brain activity patterns at the neuronal level. Our present work contributes to the understanding of NSW as a correlate of VWM capacity and demonstrates that this component is neurophysiologically plastic through training with simple video games, which are modifiable in various aspects such as difficulty, visual characteristics, fun factors, motivation, or interest in training, depending on the variables to be measured.

## Figures and Tables

**Table 1:** Accuracy in the change detection task. Both groups presented an increase in the accuracy of the 2-square set size and a decrease in the accuracy of the 8-square set. M = mean, SD = standard deviation.

	VWM Group						RT Group					
Set Size	Pre-		Mid-		Post-		Pre-		Mid-		Post-	
	M	SD	M	SD	M	SD	M	SD	M	SD	M	SD
2	96.7	3.7	95.8	2.3	98	1.4	95.8	3.8	95.1	4.1	98	2
4	86.4	9.5	89.8	3.7	88.2	5.5	86.7	4.1	89.1	6.6	86.4	6.1
6	75.6	8.9	79.8	5.9	79.6	5.7	72.4	14.6	76.7	10.4	74.4	5.8
8	69.1	10.7	73.6	7.4	66	9.3	69.8	4.3	70.4	10.6	63.6	9.8

**Table 2:** Hit Reaction Time (ms) in the change detection task. Lower mean hit RT are observable on smaller set sizes and on post-training session. M = Mean, SD = Standard Deviation.

	VWM Group						RT Group					
Set Size	Pre-		Mid-		Post-		Pre-		Mid-		Post-	
	M	SD	M	SD	M	SD	M	SD	M	SD	M	SD
2	2.365	0.2	2.417	0.2	2.336	0.1	2.553	0.5	2.364	0.1	2.448	0.1
4	2.534	0.2	2.663	0.4	2.450	0.2	2.612	0.4	2.430	0.1	2.415	0.1
6	2.665	0.3	2.575	0.2	2.504	02	2.696	0.4	2.492	0.1	2.450	0.1
8	2.650	0.3	2.693	0.3	2.547	0.2	2.648	0.3	2.564	0.1	2.473	0.1

**Table 3:** Correct rejection reaction time (ms) in the change detection task. The VWM group shows lower mean RTs on smaller set sizes, while the RT group tends to behave similarly in the sets. M = mean, SD = standard deviation.

	VWM Group						RT Group					
Set Size	Pre-		Mid-		Post-		Pre-		Mid-		Post-	
	M	SD	M	SD	M	SD	M	SD	M	SD	M	SD
2	2.426	0.2	2.417	0.2	2.347	0.1	2.595	0.3	2.363	0.1	2.318	0.1
4	2.596	0.2	2.491	0.2	2.428	0.1	2.587	0.3	2.441	0.1	2.416	0.1
6	2.727	0.3	2.551	0.1	2.517	0.1	2.705	0.4	2.536	0.2	2.513	0.2
8	2.759	0.4	2.648	0.3	2.559	0.2	2.642	0.2	2.498	0.1	2.515	0.2

**Table 4:** Cowan’s K values in the change detection task. A significant decrease in the 8-square array Cowan’s K, as well as a significant increase in the Cowan’s K values in the 2-square array trials of both groups. M = mean, SD = standard deviation.

	VWM Group						RT Group					
Set Size	Pre-		Mid-		Post-		Pre-		Mid-		Post-	
	M	SD	M	SD	M	SD	M	SD	M	SD	M	SD
2	1.87	0.1	1.83	0.1	1.92	0.1	1.83	0.1	1.80	0.2	1.92	0.1
4	2.92	0.7	3.18	0.3	3.01	0.4	2.93	0.3	3.13	0.5	2.92	0.5
6	3.07	1.1	3.57	0.7	3.54	0.6	2.69	1.8	3.2	1.2	2.93	0.6
8	3.06	1.7	3.77	1.9	2.56	1.5	3.16	0.7	3.27	1.7	2.17	1.6

**Table 5:** NSW mean voltage (µV) for the 480–900 ms time window. Greater negative voltages are progressively observed as the set size increases during the pre-training session. M = mean, SD = standard deviation.

	VWM Group						RT Group					
Set Size	Pre-		Mid-		Post-		Pre-		Mid-		Post-	
	M	SD	M	SD	M	SD	M	SD	M	SD	M	SD
2	0.14	0.6	−0.46	0.9	−0.16	0.2	−0.29	0.8	−0.24	0.7	−0.56	0.5
4	−0.65	0.4	−0.44	0.8	−0.19	0.5	−0.69	0.7	−0.82	0.9	−0.81	0.7
6	−0.66	0.5	−0.81	0.8	−0.47	0.7	−1.20	1.3	−0.92	1.2	−0.65	0.9
8	−0.88	0.6	−0.67	1.3	−0.48	0.5	−1.41	0.6	−1.19	1.3	−0.48	0.9

**Table 6:** NSW epochs features classification on different time instant comparisons (480–900 ms). F1 scores obtained by the DT.

Group	VWM Group				RT Group	
Set Size	Pre-, Mid-	Pre-, Post-	Mid-, Post-	Pre-, Mid-	Pre-, Post-	Mid-, Post-
2	0.65	0.73	0.37	0.53	0.60	0.60
4	0.66	0.37	0.63	0.68	0.66	0.69
6	0.71	0.55	0.59	0.53	0.68	0.62
8	0.67	0.67	0.53	0.54	0.61	0.59

**Table 7:** NSW epochs features classification on different evaluation sessions (500–1200 ms). F1 scores obtained by the DT.

Group	VWM Group				RT Group	
Set Size	Pre-, Mid-	Pre-, Post-	Mid-, Post-	Pre-, Mid-	Pre-, Post-	Mid-, Post-
2	0.70	0.72	0.56	0.41	0.42	0.70
4	0.67	0.67	0.68	0.40	0.56	0.67
6	0.67	0.66	0.56	0.65	0.62	0.59
8	0.64	0.61	0.53	0.58	0.57	0.61

**Table 8:** NSW epochs features classification of groups (480–900 ms). F1 scores obtained by the DT. The F1 score indicates how differentiable the features are between the groups for each given time instant.

Set Size	Pre-	Mid-	Post-
2	0.58	0.51	0.67
4	0.46	0.66	0.58
6	0.62	0.50	0.66
8	0.63	0.71	0.62

**Table 9:** NSW epochs features classification of groups (500–1200 ms). F1 scores obtained by the DT. The F1 score indicates how differentiable the features are between the groups for each given time instant.

Set Size	Pre-	Mid-	Post-
2	0.55	0.71	0.47
4	0.54	0.69	0.55
6	0.61	0.66	0.62
8	0.53	0.60	0.60

## Data Availability

The original contributions presented in this study are included in the article/Supplementary Materials. Further inquiries can be directed to the corresponding author.

## Supplementary Materials

The following supporting information can be downloaded at https://www.mdpi.com/article/10.3390/brainsci15020153/s1, Figure S1: VWM group ERP’s obtained in function of the set size for a change detection task before the training (pre-training). NSW for different set sizes are shown with different color in the gray section of the box; Figure S2: VWM group ERP’s obtained in function of the set size for a change detection task in the middle of the training (mid-training). NSW for different set sizes are shown with different color in the gray section of the box; Figure S3: VWM group ERP’s obtained in function of the set size for a change detection task after the training (post-training). NSW for different set sizes are shown with different color in the gray section of the box; Figure S4: RT group ERP’s obtained in function of the set size for a change detection task before the training (pre-training). NSW for different set sizes are shown with different color in the gray section of the box; Figure S5: RT group ERP’s obtained in function of the set size for a change detection task in the middle of the training (mid-training). NSW for different set sizes are shown with different color in the gray section of the box; Figure S6: RT group ERP’s obtained in function of the set size for a change detection task after the training (post-training). NSW for different set sizes are shown with different color in the gray section of the box; Figure S7: VWM group ERP’s obtained in function of training stage for a change detection task of 2 squares. NSW for the three training stages are shown with different color in the gray section of the box; Figure S8: VWM group ERP’s obtained in function of training stage for a change detection task of 4 squares. NSW for the three training stages are shown with different color in the gray section of the box; Figure S9: VWM group ERP’s obtained in function of training stage for a change detection task of 6 squares. NSW for the three training stages are shown with different color in the gray section of the box; Figure S10: VWM group ERP’s obtained in function of training stage for a change detection task of 8 squares. NSW for the three training stages are shown with different color in the gray section of the box; Figure S11: RT group ERP’s obtained in function of training stage for a change detection task of 2 squares. NSW for the three training stages are shown with different color in the gray section of the box; Figure S12: RT group ERP’s obtained in function of training stage for a change detection task of 4 squares. NSW for the three training stages are shown with different color in the gray section of the box; Figure S13: RT group ERP’s obtained in function of training stage for a change detection task of 6 squares. NSW for the three training stages are shown with different color in the gray section of the box; Figure S14: RT group ERP’s obtained in function of training stage for a change detection task of 8 squares. NSW for the three training stages are shown with different color in the gray section of the box.
