# Supplementary material for: A Pilot Study on Video Game Training Effects on Visual Working Memory: Behavioral and Neural Insights
Source: Brain Sci. 2025 Feb 4;15(2):153. doi: 10.3390/brainsci15020153 (PMC11852622; doi:10.3390/brainsci15020153)
Supplement: Supplementary file 1 [file brainsci-15-00153-s001.zip › Figure S1-S6.pdf]

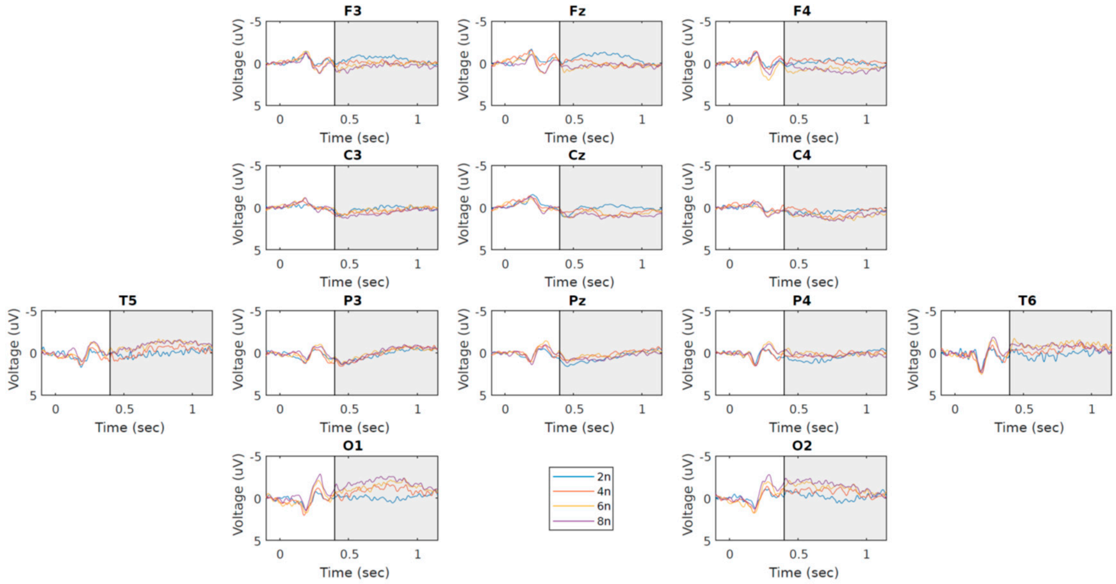

Figure S1: VWM group ERP's obtained in function of the set size for a change detection task before the training (pre-training). NSW for different set sizes are shown with different color in the gray section of the box.

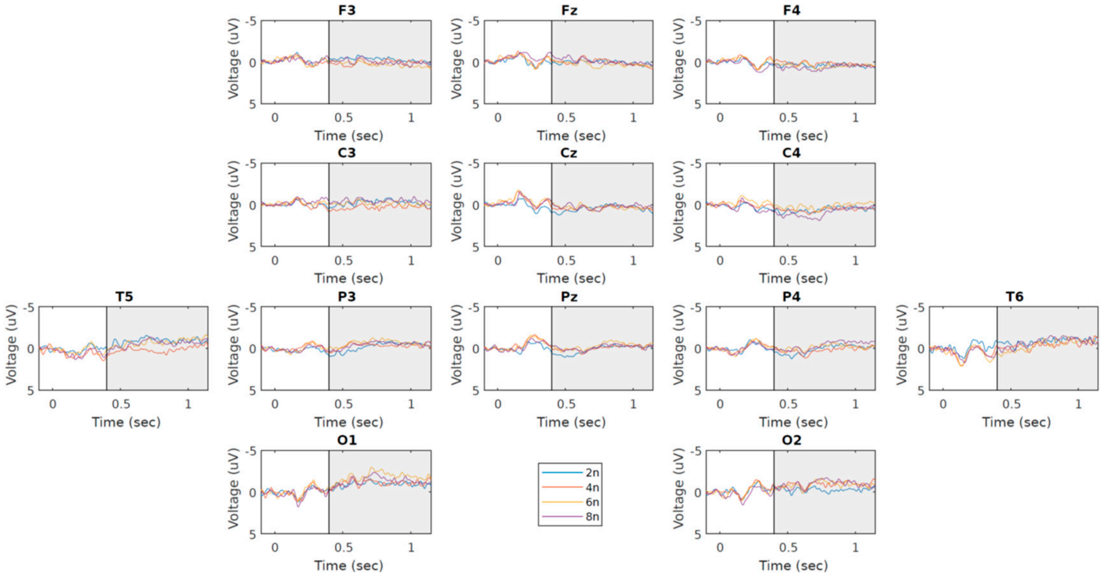

Figure S2: VWM group ERP's obtained in function of the set size for a change detection task in the middle of the training (mid-training). NSW for different set sizes are shown with different color in the gray section of the box.

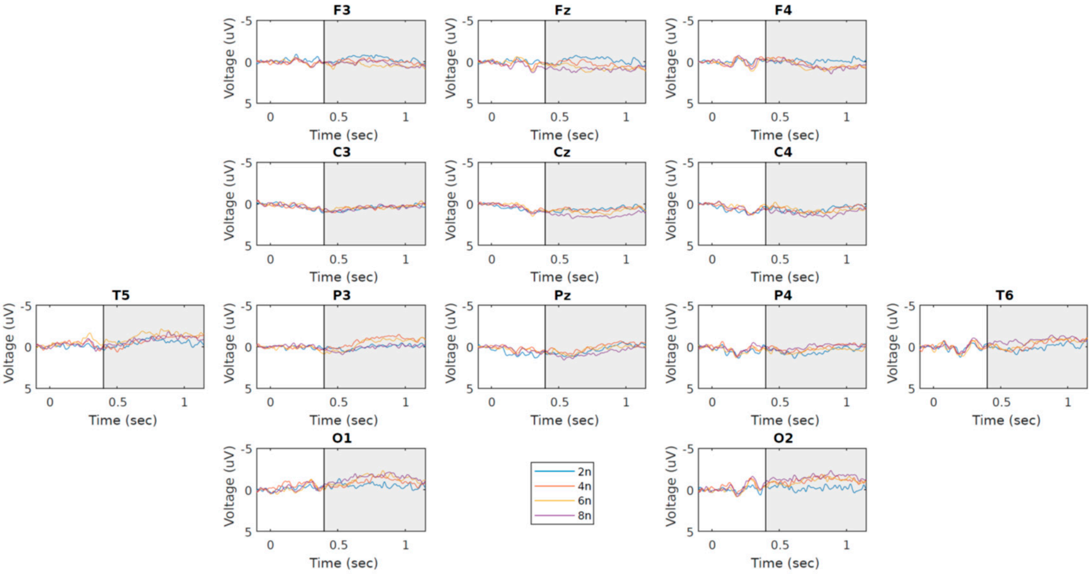

Figure S3: VWM group ERP's obtained in function of the set size for a change detection task after the training (post-training). NSW for different set sizes are shown with different color in the gray section of the box.

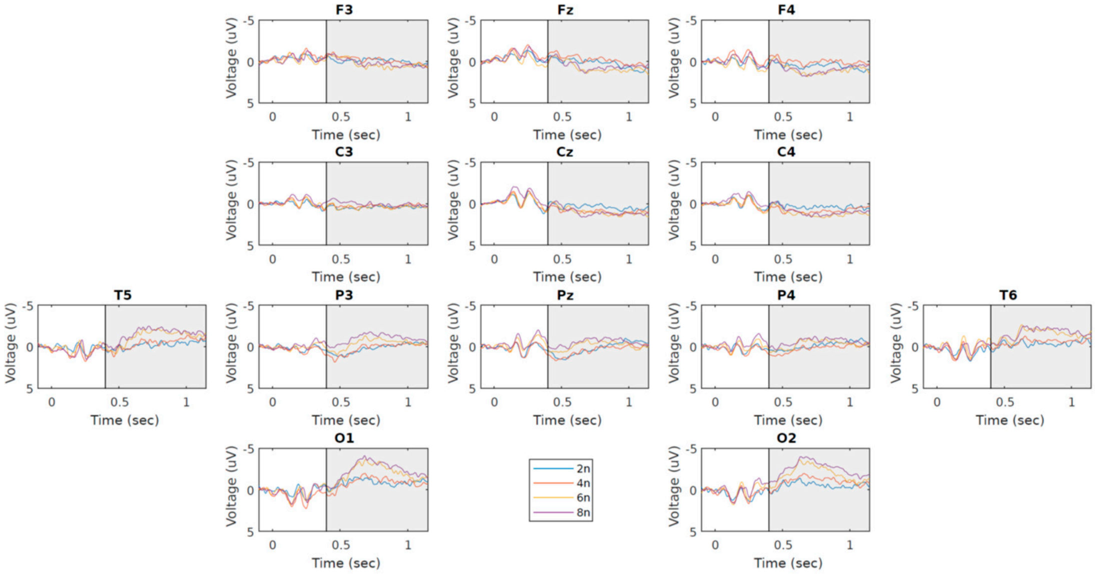

Figure S4: RT group ERP's obtained in function of the set size for a change detection task before the training (pre-training). NSW for different set sizes are shown with different color in the gray section of the box.

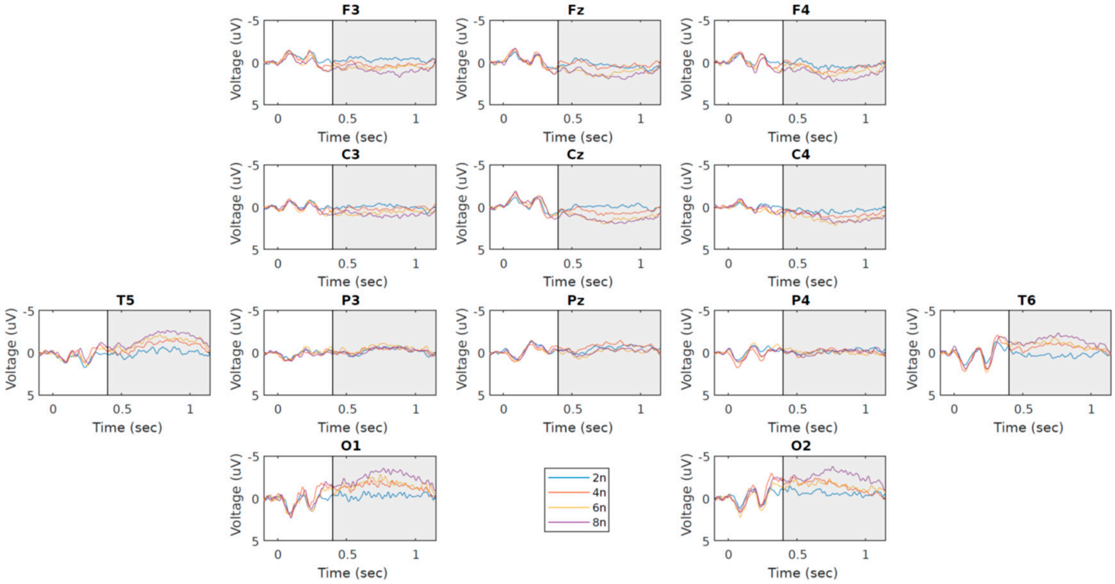

Figure S5: RT group ERP's obtained in function of the set size for a change detection task in the middle of the training (mid-training). NSW for different set sizes are shown with different color in the gray section of the box.

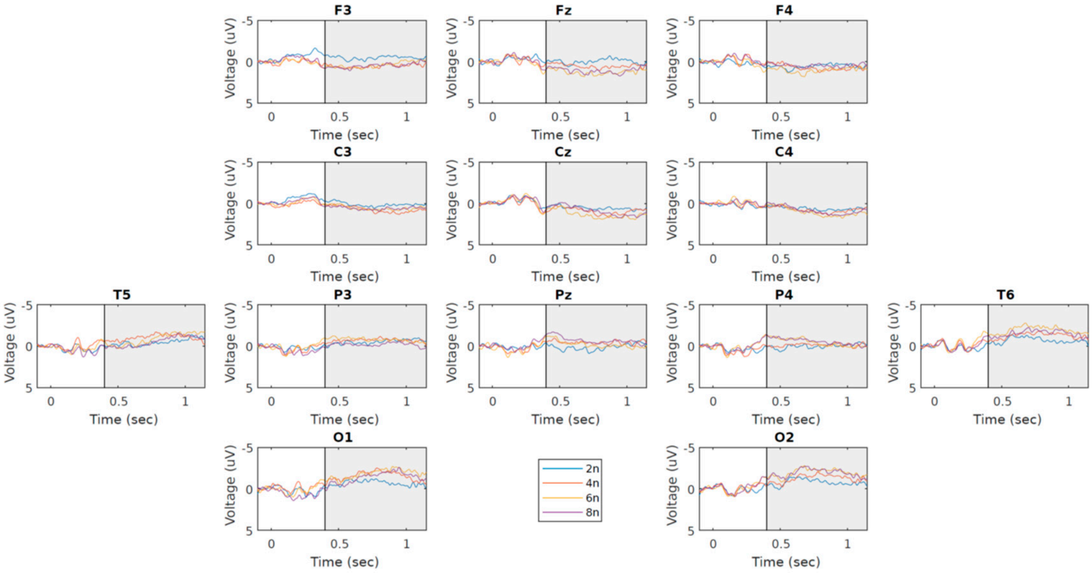

Figure S6: RT group ERP's obtained in function of the set size for a change detection task after the training (post-training). NSW for different set sizes are shown with different color in the gray section of the box.
