# Supplementary material for: A Pilot Study on Video Game Training Effects on Visual Working Memory: Behavioral and Neural Insights
Source: Brain Sci. 2025 Feb 4;15(2):153. doi: 10.3390/brainsci15020153 (PMC11852622; doi:10.3390/brainsci15020153)
Supplement: Supplementary file 1 [file brainsci-15-00153-s001.zip › Figure S7-S14.pdf]

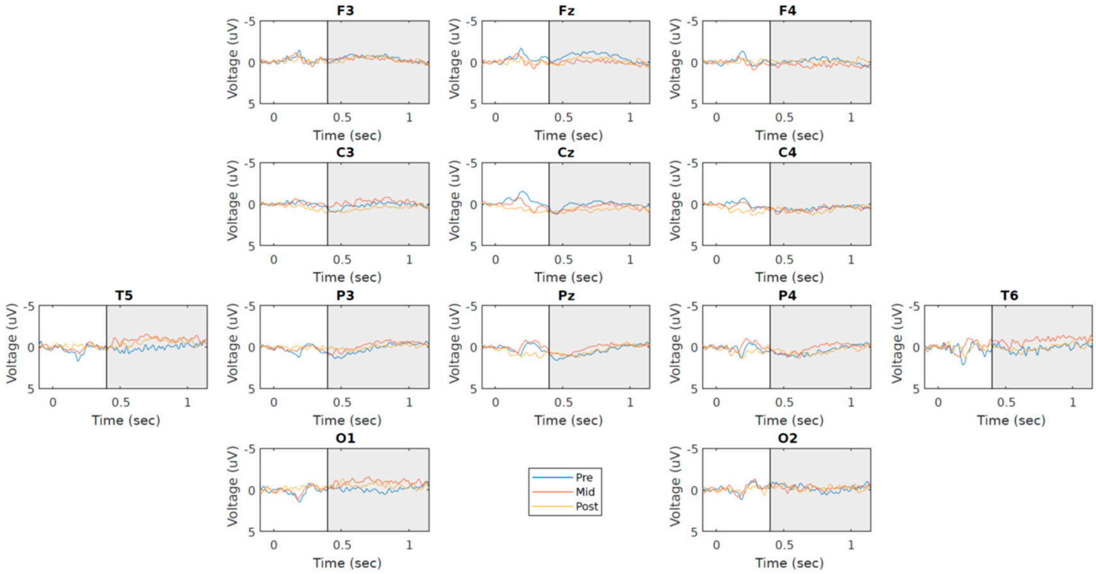

Figure S7: VWM group ERP's obtained in function of training stage for a change detection task of 2 squares. NSW for the three training stages are shown with different color in the gray section of the box.

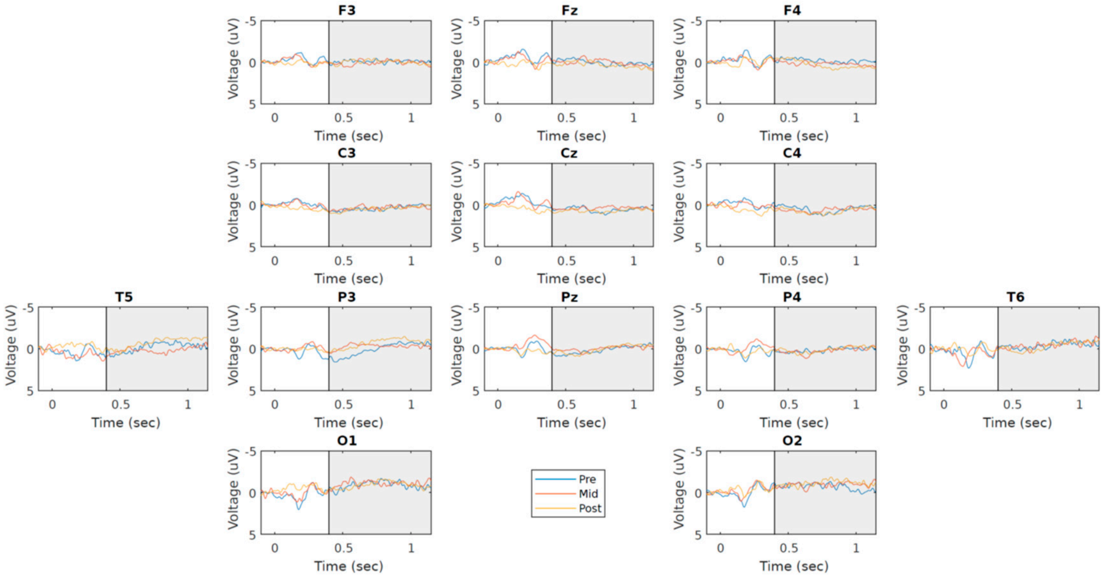

Figure S8: VWM group ERP's obtained in function of training stage for a change detection task of 4 squares. NSW for the three training stages are shown with different color in the gray section of the box.

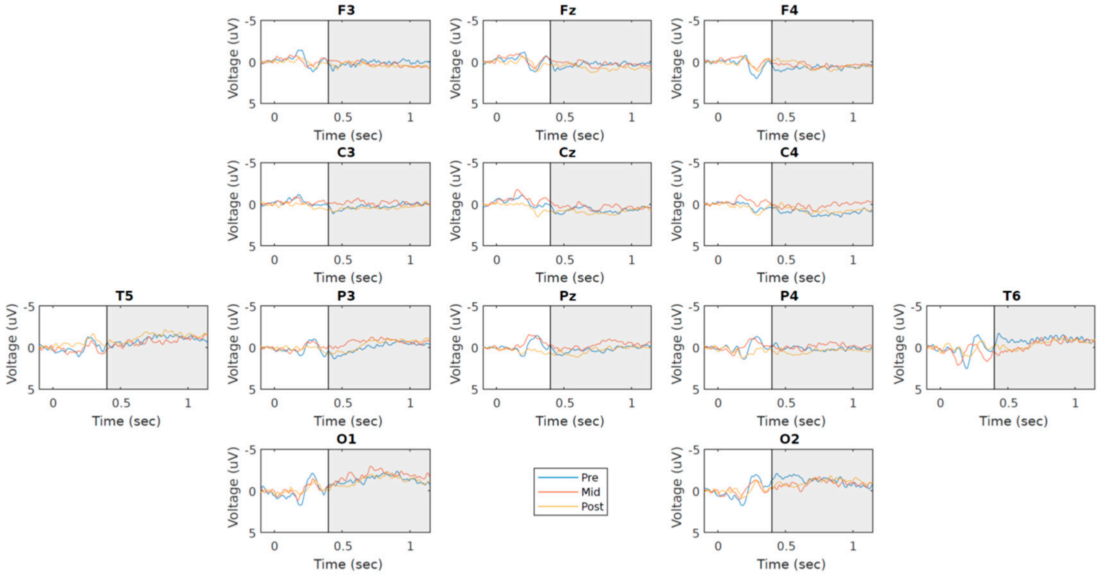

Figure S9: VWM group ERP's obtained in function of training stage for a change detection task of 6 squares. NSW for the three training stages are shown with different color in the gray section of the box.

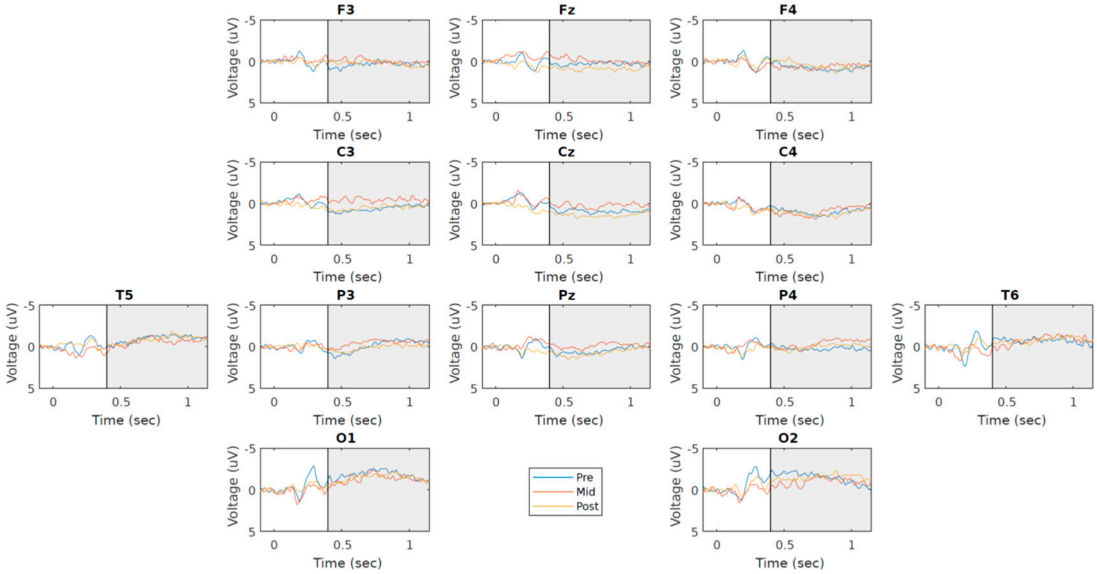

Figure S10: VWM group ERP's obtained in function of training stage for a change detection task of 8 squares. NSW for the three training stages are shown with different color in the gray section of the box.

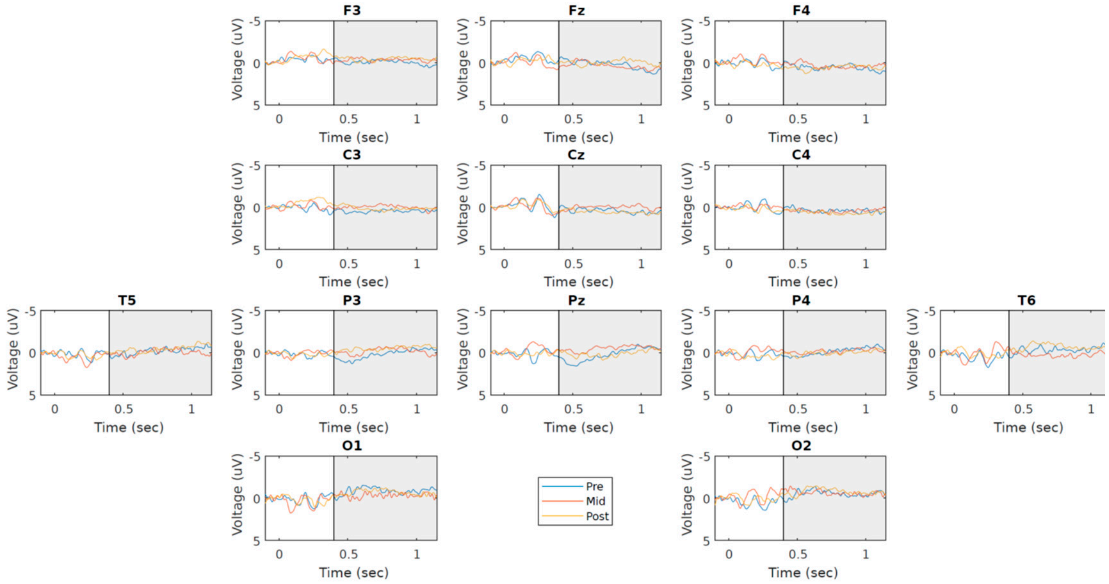

Figure S11: RT group ERP's obtained in function of training stage for a change detection task of 2 squares. NSW for the three training stages are shown with different color in the gray section of the box.

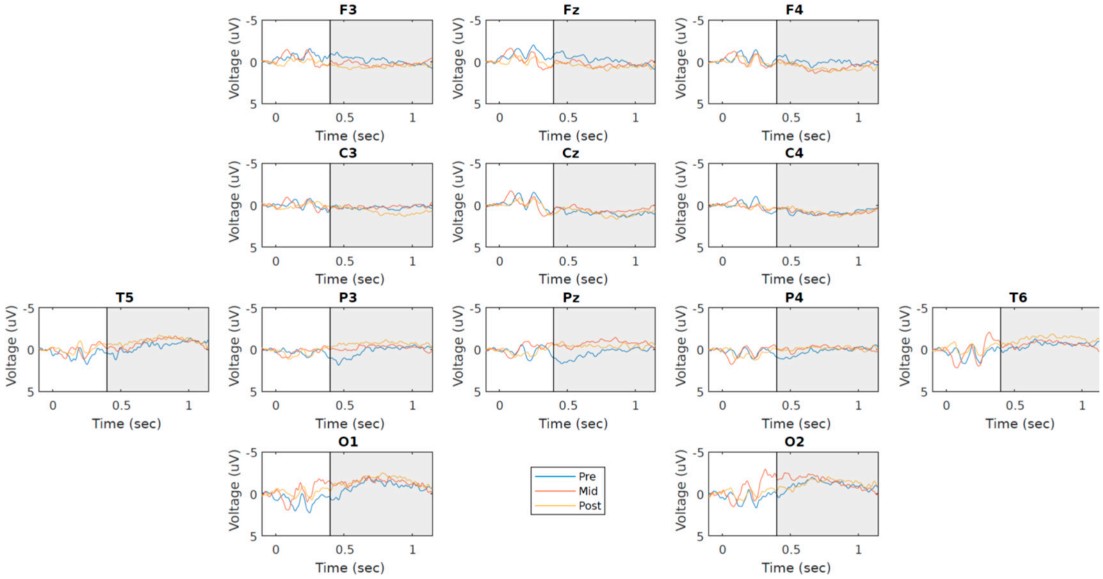

Figure S12: RT group ERP's obtained in function of training stage for a change detection task of 4 squares. NSW for the three training stages are shown with different color in the gray section of the box.

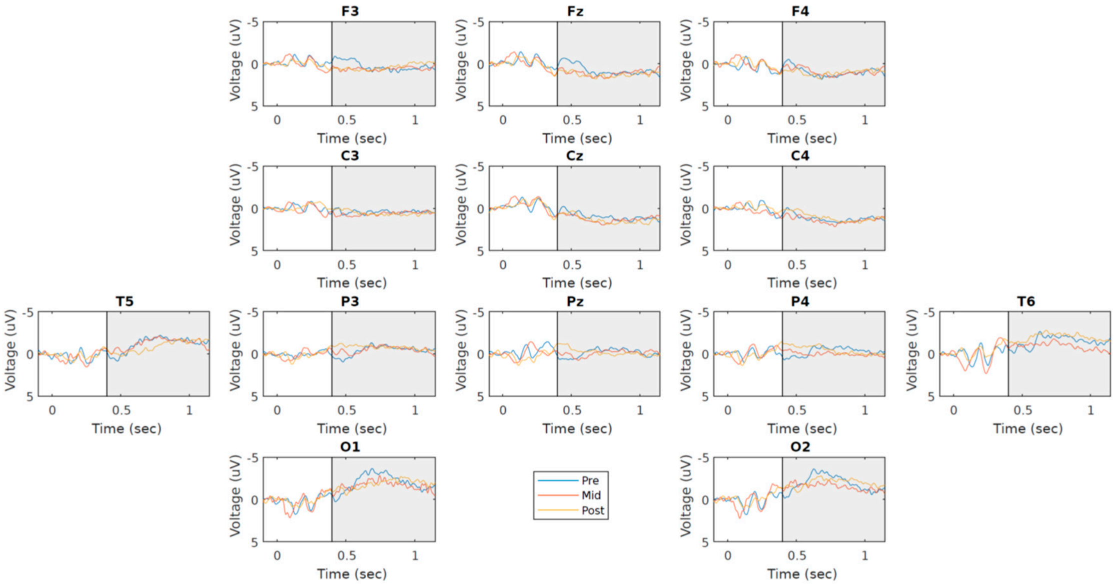

Figure S13: RT group ERP's obtained in function of training stage for a change detection task of 6 squares. NSW for the three training stages are shown with different color in the gray section of the box.

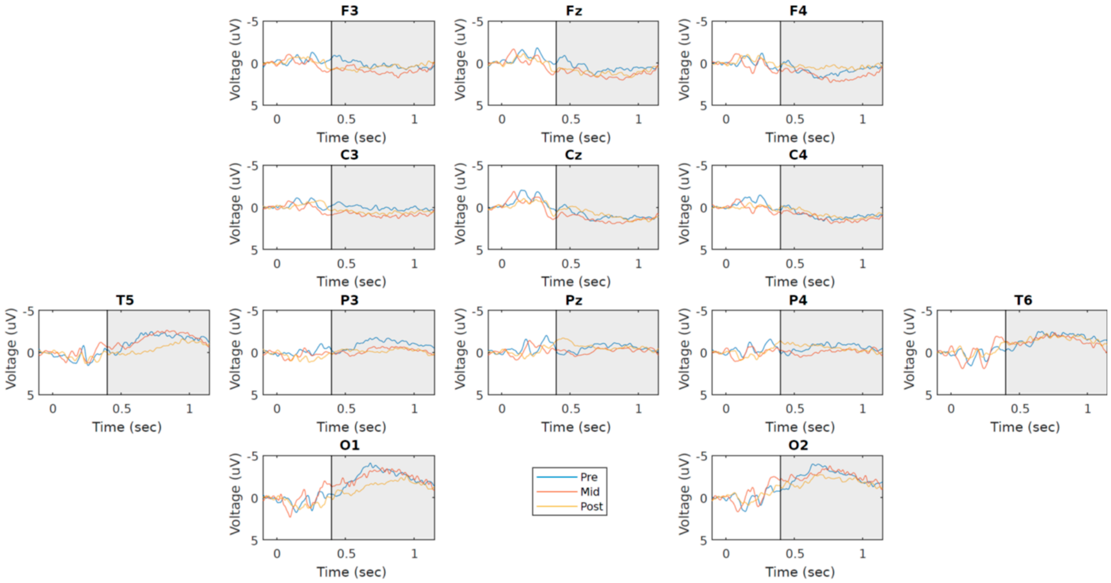

Figure S14: RT group ERP's obtained in function of training stage for a change detection task of 8 squares. NSW for the three training stages are shown with different color in the gray section of the box.
